# Synthesis, Biological Evaluation, and Computational Analysis of 1,4-Naphthoquinone Derivatives as Inhibitors of the Sodium-Dependent NADH:Ubiquinone Oxidoreductase (NQR) in *Vibrio cholerae*

**DOI:** 10.3390/ijms27031198

**Published:** 2026-01-24

**Authors:** Zachary J. Liveris, Ming Yuan, Yuyao Hu, Jennifer M. Sorescu, Karina Tuz, Oscar X. Juárez, Daniel P. Becker

**Affiliations:** 1 Department of Chemistry and Biochemistry, Loyola University Chicago, 1032 West Sheridan Road, Chicago, IL 60660, USA; zliveris@luc.edu; 2 Department of Biological Sciences, Illinois Institute of Technology, Chicago, IL 60616, USA; myuan0909@gmail.com (M.Y.); yhu68@hawk.illinoistech.edu (Y.H.); jsorescu@hawk.illinoistech.edu (J.M.S.); ktuz@illinoistech.edu (K.T.)

**Keywords:** sodium-dependent NADH:ubiquinone oxidoreductase, NQR, naphthoquinone, antibiotic, cholera

## Abstract

The therapeutic efficacy of antibiotics has been significant in extending human life expectancy by combating virulent bacterial infections. Nevertheless, multidrug-resistant (MDR) microorganisms remain a global crisis as these bacteria have developed resistance to conventional antibacterial agents. An unexplored antibiotic target found exclusively in bacteria is the Na^+^-translocating NADH:ubiquinone oxidoreductase (NQR), which is an indispensable membrane-bound bacterial enzyme complex that enables cellular functionality and is present in many infectious bacterial species, including *Vibrio cholerae* and *H. influenzae*. NQR serves as an essential complex in the bacterial electron transport chain (ETC) and operates as a highly conserved primary Na^+^ pump that drives many bioenergetic functions. This six-subunit protein shuttles electrons from NADH to ubiquinone, which drives the translocation of Na^+^ ions and creates a gradient that provides the driving force for various cellular processes. We have synthesized and evaluated a series of 1,4-naphthoquinones that exhibit high potency against NQR with minimal cytotoxicity and potential to serve as new, NQR-targeting antibacterial agents for use against *V. cholerae*.

## 1. Introduction

Cholera is an acute and often deadly gastrointestinal infection caused by the marine aerobic Gram-negative bacteria *Vibrio cholerae*, which colonizes the small intestine and has led to numerous documented epidemics since 1817 [1]. Cholera is contracted through the consumption of unsanitary drinking water, typically in underdeveloped countries. Although antibiotics are an effective treatment, the World Health Organization (WHO) has underscored the exponential rate of antibiotic resistance to this bacterium [1]. Once an individual is infected, *V. cholerae* adheres to the epithelial cells that line the duodenum of the host, aided by the toxin-coregulated pilus (TCP) [2,3]. The pathogen then expresses and secretes an oligomeric protein, the cholera toxin (CT), which binds to the ganglioside receptor GM1 lining the epithelial cells [2,3,4]. This in turn leads to the overproduction of cyclic AMP (cAMP) in the host cells, resulting in excessive water and electrolyte secretion [4].

NQR is a sodium-dependent NADH:ubiquinone oxidoreductase, comprising six subunits (NqrA through NqrF), and is an essential respiratory enzyme that functions as a primary Na^+^ pump utilizing ubiquinone (Q) and NADH as primary substrates [5]. This electron transport chain (ETC) enzyme is essential for prokaryotic cell survival, and is found in many pathogenic proteobacteria, including *V. cholerae* [6]. The human genome does not contain any homologous proteins, thus establishing the attractiveness of this potential antibiotic target in avoiding mechanism-based toxicity. Through its sodium pumping activity, NQR heads the metabolic respiratory chain machinery that sustains a number of critical cellular functions, including ATP synthesis, pH regulation, drug efflux, and expression of the transcriptional activator ToxT, which is responsible for regulating the transcription of both CT and TCP genes [3,7,8]. NQR operates by shuttling electrons from NADH to the ubiquinone substrate, using six cofactors, and the energy derived from this process drives the translocation of Na^+^ ions across the plasma membrane.

**Figure 1:**
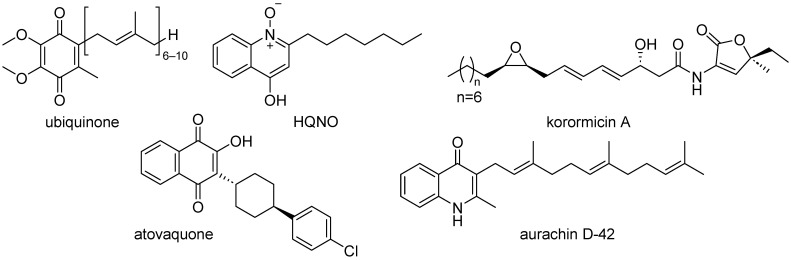
Structures of the NQR substrate ubiquinone, HQNO, korormicin A, atovaquone, and aurachin D-42.

Masuya and coworkers have explored the *N*-terminal region of NQR using a sequential conjugation technique with korormicin A as a ligand to deliver probes to monitor dynamic structural changes in the cytoplasmic *N*-terminal region of the NqrB subunit. The NqrB subunit plays an important role in the terminal ubiquinone reduction in the NQR electron transport chain, and is also the active site target of specific inhibitors [9]. Using cryo-EM structures and isothermal titration calorimetry to elucidate photoaffinity labeling studies using photoreactive korormicin, Murai also used cryo-EM to demonstrate that an NqrB-G141A mutant, which has some resistance to korormicin A, is mediated by a conformationally altered binding cavity that leads to decreased potency by korormicin A [10]. NQR is the main entry point of electrons into the respiratory chain [11]. The NQR binding site for ubiquinone is hypothesized to be the binding site for inhibitors, including ubiquinone analogs—such as HQNO—as well as korormicin A (Figure 1). Both of these respiratory inhibitors have been shown to effectively inhibit NQR, whereas toxicity in humans by these derivatives at high dosages is a result of off-target cytochrome *bc*_1_ inhibition [7,12,13]. In this work, we are evaluating a first generation of 1,4-naphthoquinones that target NQR as potential antibiotics. Naphthoquinones are druggable scaffolds and have been employed in approved drugs to treat cancer, osteoarthritis, blood clotting, and malaria [14].

NQR is a promising target for the discovery of new antibiotics [15], and we have shown that clofazimine can be repurposed to treat cholera, offering a novel class of antibiotics that target NQR [16]. Seeking other chemical templates, we screened several ubiquinone-like compounds against the target enzyme NQR and discovered that atovaquone (Figure 1) is an inhibitor of NQR (IC_50_ = 8.0 µM). This clinically approved antimalarial drug has been observed to interfere with ubiquinone functionality in the bacterial respiratory cycle, more specifically through selective inhibition of the malarial parasite cytochrome *bc*_1_ complex [17,18]. Using atovaquone as a lead scaffold, we report here—for the first time—the design, synthesis, and characterization of a series of novel 1,4-naphthoquinone derivatives that potently inhibit NQR at low micromolar concentrations. These compounds exhibit high inhibitory potency while displaying minimal cytotoxicity, highlighting their potential as a new, non-toxic class of NQR-targeting antibacterial agents. To our knowledge, this work represents the first systematic synthesis and evaluation of 1,4-naphthoquinone-based NQR inhibitors, establishing a new chemical framework for antibiotic development against *Vibrio cholerae*.

## 2. Results

### 2.1. Synthesis of 1,4-Naphthoquinone Inhibitors

**Scheme 1:**
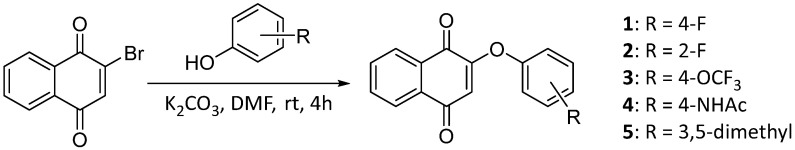
Synthesis of 2-phenoxy-naphthoquinones from 2-bromo-naphthoquinone.

To optimize NQR inhibition starting with atovaquone, we focused initially on modulating positions 2 and 3 on the 1,4-naphthoquinone core. The preparation of the first set of naphthoquinone analogs, illustrated in Scheme 1, involves the single installment of phenoxy sidechains on C2 through nucleophilic substitution of 2-bromo-naphthoquinone using the substituted phenols, resulting in the desired 2-phenoxy-naphthoquinones [19]. We noted some instability associated with naphthoquinones **1**–**3** bearing electron-withdrawing substituents, presumably as a result of addition and elimination of the phenoxy moiety as a presumed leaving group [19], which was not an issue with electron-rich phenolic substituents (**4**–**5**).

**Scheme 2:**
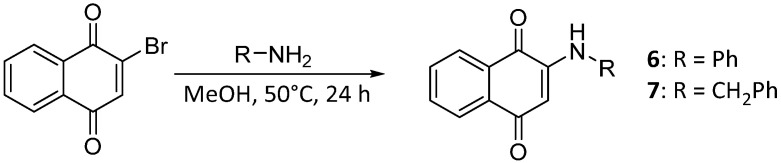
Synthesis of 2-amino-naphthoquinones.

To further enhance the stability associated with the phenoxy analogs and explore the effect of heteroatomic substituents on the quinone ring, aniline and benzylamine were added, affording amino-substituted naphthoquinones **6** and **7** (Scheme 2).

**Scheme 3:**
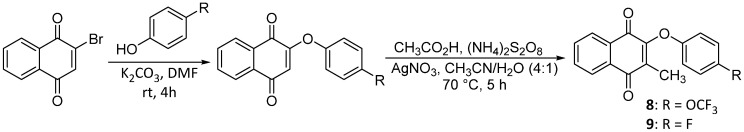
Synthesis of 3-methyl-2-phenoxy-naphthoquinones via nucleophilic substitution and radical-Hundsdiecker decarboxylation.

We pursued alkylation at C3 both to explore the effect on potency while retaining the phenoxy unit at C2, and to enhance the stability relative to nucleophilic attack on the naphthoquinone functionality. The first route that was explored was through direct methylation of 2-bromo-naphthoquinone using a modified radical-Hundsdiecker decarboxylation, followed by nucleophilic substitution with the corresponding phenol [19,20]. This approach was initially unsuccessful; however, reversing the methylation and substitution steps afforded the desired product, as shown in Scheme 3, yielding 3-methyl-2-phenoxy-naphthoquinones.

**Scheme 4:**
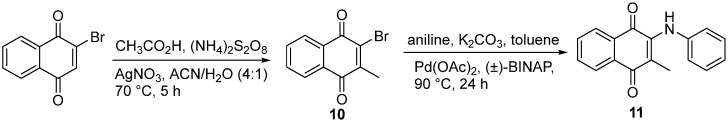
Synthesis of 2-(phenylamino)-3-methyl-naphthoquinone.

The next set of naphthoquinone candidates retained the C3-methyl substituent while incorporating an aniline moiety at C2, and the approach to access these amino-naphthoquinones is illustrated in Scheme 4. The Hundsdiecker decarboxylation reaction was used to prepare intermediate **10**, followed by a palladium-catalyzed C-N bond formation in the presence of aniline and racemic BINAP to produce the desired methylated phenylamino-naphthoquinone **11** [21].

**Scheme 5:**
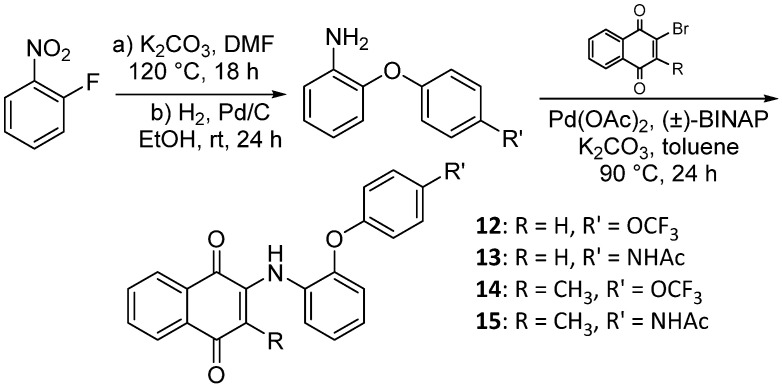
Synthesis of 2-o-aryletheranilino-naphthoquinones.

Expanding the aniline substituent at C2, we assembled two separate *o*-arylether anilines to be coupled onto the 1,4-naphthoquinone scaffold (Scheme 5). The synthesis of the arylether anilines coupling partners commenced by subjecting 2-fluoro-nitrobenzene to a nucleophilic aromatic substitution using the corresponding phenol, followed by nitroarene hydrogenation, affording the substituted aniline [22]. Subsequently, the coupling partners were installed onto both 2-bromo-naphthoquinone and 2-bromo-3-methyl-bromonaphthoquinone (**10**) using a palladium-catalyzed C-N bond formation reaction [21]. The four target analogs (**12**–**15**) were constructed in order to differentiate the impact on inhibitory potency associated with the C3-methyl group.

### 2.2. Biological Evaluation

#### 2.2.1. Activity and Toxicity Screening

The synthesized compounds were tested for their inhibitory activity on *V. cholerae* NQR at 10 and 50 μm, and were assessed for their toxicity against HeLa cells and HFF-1 cells at 10 μM. As shown in Table 1, 1,4-naphthoquinone itself is a potent inhibitor of NQR but also shows high toxicity against human cells. The main aim of this project is to use the naphthoquinone scaffold to design more potent NQR inhibitors with low toxicity that can be used as antibiotics to treat cholera. The compounds that were synthesized showed an improved toxicity profile, while aryloxy naphthoquinones **2**, **4**, **5**, and **8** still showed significant toxicity (>40%). Most of the other compounds showed improved toxicities <30%, with naphthoquinones **1, 12**, and **14** showing excellent low toxicities (≤10%). In particular, diaryl ethers **12** and **14** showed negligible toxicity. Regarding potency of inhibition, some trends can be observed. There were no significant differences for the two R_1_ substituents, H or CH_3_, but the aryl R_2_ substituent greatly modulated inhibitory potency. For instance, the inhibitory activity of fluorophenoxy compounds **1**, **9**, and **2** was modest, with inhibitions of 20–30% at 10 μM. Interestingly, while fluorophenyloxy-naphthoquinones **1** and **2** showed similar potencies, their toxicity was vastly different, indicating that the position of the fluoride atom in the phenoxy ring (ortho vs. para) is critical to developing safe compounds. The inhibitory activity of fluorophenoxy compounds was comparable to inhibitors of a similar size, such as trifluoromethoxyphenoxy compounds (**8** and **3**), dimethylphenoxy (**5**), and acetamidophenoxy (**4**). Trifluoromethoxyphenoxyphenylamino compounds **12** and **14** were potent, with inhibition >72% at 10 μM. Moreover, these inhibitors also showed the lowest toxicity of all compounds tested (5%). The inhibitory activity of **12** and **14** can be attributed to the hydrophobicity of the R_2_ group, as the inhibitory activity is decreased when a relatively hydrophilic functional group is introduced, such as the acetamidophenoxy derivatives **13** and **15**, which showed less than half the inhibitory activity at 10 μM. Moreover, the effect of the geometry of the R_2_ group was also evident as the position of the trifluoromethoxyphenoxy moiety in the ortho position resulted in the more potent diaryl ether naphthoquinones **12** and **14**, while in the para position (**16**), this diminished the inhibitory activity by approximately one-third. Moreover, the trifluoromethoxyphenoxy moiety significantly enhances the inhibitory potency in contrast to the ar(alkyl)aminonaphthoquinones (**6**, **11**, and **7**), which lack this functional group and do not show potent inhibitory profiles.

#### 2.2.2. IC_50_ Measurements

To further characterize the potency of the best inhibitors, IC_50_ measurements were carried out on select analogs, measuring the activity of the purified NQR complex at different concentrations of the inhibitor. The IC_50_ values (Table 2) are consistent with the structural analysis discussed above, with compounds **12** and **14** showing IC_50_ values of 12–16 μM, while the related but structurally different compound **16** showed 3 times weaker IC_50_. Moreover, compound **6**, which lacks the trifluoromethoxyphenoxy group, is a very weak inhibitor of NQR (Table 2).

#### 2.2.3. Molecular Docking

**Figure 2:**
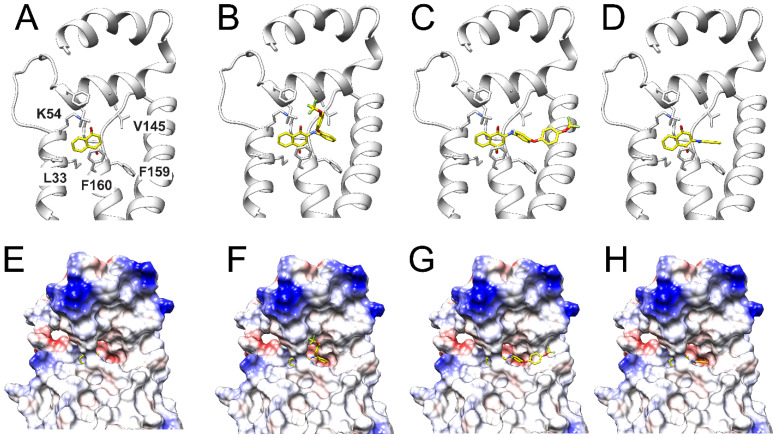
Docking of the compounds (yellow) tested in this work. Naphthoquinone (**A**,**E**), **12** (**B**,**F**), **16** (**C**,**G**), and **6** (**D**,**H**) in the proposed ubiquinone binding site of NQR. The figure shows the ribbon representation of subunit B structure (**A**–**D**) and the surface of the subunit colored by Coulombic charges (**E**–**H**). Red, negative; blue, positive; and white, uncharged.

To understand the molecular basis for the inhibition of the compounds that were synthesized, molecular docking analysis was carried out. The NQR cryo-EM structure obtained by our group (PDB ID: 8EVU), which closely resembles other published structures [23,24] with RMSD values <1 Å, was used to predict poses of inhibitors into the substrate binding site. The docking analysis was performed on the site of the protein that carries the tightly bound ubiquinone molecule, in subunit B [24]. Our data show that naphthoquinone interacts with the ubiquinone binding site by making π-π contacts with Phe160 and a hydrogen bond with Lys54 (Figure 2A). These residues have been reported as important for binding the ubiquinone substrate into the site and facilitating the binding of other inhibitors, such as HQNO and the quinolone Aurachin-D [24]. Compound **12**, our most potent inhibitor thus far, is also able to establish these interactions, and it is further stabilized in the site through hydrophobic interactions of the trifluoromethoxyphenoxyphenylamino moiety with the entry pathway of ubiquinone in the site (Figure 2B,F). Interestingly, the binding site has a curvature that allows the benzoquinone head of ubiquinone to enter the site, leaving the isoprene tail of the molecule facing the membrane environment. The geometry of this curvature is closely followed by compound **12** (Figure 2F), consistent with its high inhibitory potency. On the other hand, the closely related compound **16**, which also appears to interact with Phe160 and Lys54 (Figure 2C), is mostly linear due to the *para* position of the trifluoromethoxyphenoxy substituent of the molecule, as discussed above, and does not fit well into the ubiquinone site (Figure 2G), explaining its lower potency. The smaller compound **6** also makes contact with the site (Figure 2D,H), but lacks other important hydrophobic contacts in the site, explaining its low potency.

## 3. Discussion

In this work, we have designed 1,4- naphthoquinone derivatives that can serve as inhibitors of the essential respiratory complex NQR. Our data show that we can obtain molecules with comparable potency to the lead naphthoquinone atovaquone, but that have negligible cellular toxicity, which represents a critical step toward the development of new antibiotics against pathogens that rely on NQR activity. Naphthoquinones are particularly relevant as scaffolds to produce new molecules with therapeutic applications, as they have been used in the development of atovaquone. Atovaquone is a 2-hydroxy naphthoquinone that has shown potent inhibitory activity against malaria, toxoplasma, and the pneumocystis respiratory cytochrome *bc*_1_ complex [25,26]. The most potent naphthoquinones **12** and **14** (IC_50_ = 12 ± 1 μM and 16 ± 3 μM, respectively) both bear an ortho-substituted trifluoromethyoxyphenyloxyphenylamino substituent in the 3 position and are more potent than the para-substituted naphthoquinone **16** (IC_50_ = 46 ± 5 μM), which is consistent with docking results and the shape of the active site. Smaller substituents in the naphthoquinone 3 position were much less potent. The potent trifluoromethoxy diaryl ethers **12** and **14** differ only in the absence (R_1_ = H for **12**) or presence (R_1_ = CH_3_ for **14**) of the 2-methyl substituent, slightly favoring the unsubstituted version, but with borderline statistical significance, demonstrating little difference between these two possibilities. The production of antibiotics that target the respiratory chain of pathogenic bacteria has been overlooked for the most part, and this work aims to provide the initial steps towards this goal.

## 4. Materials and Methods

### 4.1. Synthesis

All reagents and solvents were purchased from commercial suppliers and used without any further purification unless specified. All synthetic reactions were maintained under an inert atmosphere of either nitrogen or argon. All reactions were monitored by both TLC and HPLC. Analytical thin-layer chromatography (TLC) was carried out on aluminum-backed silica gel plates (200 μm) and visualized under UV light at 254 nm. Flash column chromatography was carried out for sample purification using a CombiFlash Rf+ automated chromatography system (Teledyne ISCO, Lincoln, NE, USA) with prepacked disposable RediSep Rf flash columns (60 Å, 40–63 μm, 230/400 mesh; Avantor/VWR International, Radnor, PA, USA) coupled to a 200–400 nm variable detector. Melting points were gathered using 90 mm capillary tubes in a MelTemp melting point apparatus and were uncorrected. Analytical HPLC was performed on all samples to determine a purity of ≥95% using an Agilent 1100 series (Agilent, Santa Clara, CA, USA) high-performance liquid chromatography system with a reversed-phase C18 column, autosampler, and a diode-array detector (DAD) employing a gradient of 90% A (5% MeCN in H_2_O) and 10% B (MeCN with 0.1% TFA) with a flow rate of 1.0 mL/min. ^1^H and ^13^C nuclear magnetic resonance (NMR) spectra were conducted on a 500 MHz Brucker spectrometer. Chemical shifts were reported in parts per million (ppm), and tetramethylsilane (TMS) was used as an internal reference. High-resolution mass spectra (HRMS) were measured on a Time-of-Flight (TOF) instrument using electrospray ionization (ESI). High-resolution mass spectra (HRMS) data were obtained at the Integrated Molecular Structure Education and Research Center (IMSERC, Northwestern University, Evanston, IL, USA). HRMS spectra were collected using a Waters Acquity I class UPLC and Xevo G2-XS QTof mass spectrometer with Waters Acquity BEH C18 column (1.7 µm, 2.1 × 50 mm) (Waters Corporation, Milford, MA, USA). Mobile phase A was 0.05% formic acid in water, and mobile phase B was 0.05% formic acid in acetonitrile; a gradient of 5 to 90% B in Mobile phase A over 5 min was applied. Acquired data were processed using MassHunter software version B.04.00. HPLC traces, ^1^H NMR spectra, ^13^C NMR spectra, and HRMS data are included in the Supplementary Materials for all new compounds (**2**, **3**, **5**, **8**, **9**, **12**, **13**, **14**, **15**, **16**).

#### 4.1.1. 2-(4-Fluorophenoxy)lnaphthalene-1,4-dione (**1**)

The title compound **1** was prepared according to the method of Prati [19], and spectra were consistent with reported values.

#### 4.1.2. 2-(2-Fluorophenoxy)-3-methylnaphthalene-1,4-dione (**2**)

A solution of 2-fluorophenol (0.42 mmol) and K_2_CO_3_ (0.42 mmol) in anhydrous DMF (8.8 mL) was stirred at room temperature for 1 h under nitrogen. Subsequently, 2-bromonaphthoquinone (100 mg, 0.42 mmol) dissolved in DMF (2 mL) was added to the reaction mixture, and the resulting solution was stirred at room temperature for 3 h under nitrogen. Afterwards, the solution was poured over ice water and placed in the freezer for 3 h, which resulted in the formation of a precipitate. The precipitate was collected by vacuum filtration and washed successively with cold water, resulting in the isolation of the title compound as an off-white clayish solid (96.1 mg, 43% yield). HPLC analysis: 99.8% AUC at 10.206 min, mp 110–111 °C. ^1^H NMR (500 MHz, CDCl_3_) δ 8.26–8.18 (m, 1H), 8.11–8.03 (m, 1H), 7.82–7.73 (m, 2H), 7.35–7.27 (m, 1H), 7.30–7.20 (m, 3H), 5.95 (d, *J* = 1.6 Hz, 1H). ^13^C NMR (126 MHz, CDCl_3_) δ 184.8, 179.3, 159.1, 153.7 (d, J_CF_ = 253 Hz), 139.7 (d J_CF_ = 12.6 Hz), 134.5, 133.7, 131.9, 131.1, 128.1 (d, J_CF_ = 7.6 Hz), 126.9, 126.3, 125.4 (d, J_CF_ = 3.8 Hz), 123.3, 117.7 (d, J_CF_ = 17.6 Hz), 113.2. HRMS (ESI): *m*/*z* calculated C_16_H_10_FO_3_^+^ for [M+H]^+^: 269.0579, found 269.0605.

#### 4.1.3. 2-(4-(Trifluoromethoxy)phenoxy)naphthalene-1,4-dione (**3**)

A solution of 4-(trifluoromethoxy)phenol (0.42 mmol) and K_2_CO_3_ (1.27 mmol) in anhydrous DMF (8.8 mL) was stirred at room temperature for 1 h under nitrogen. Subsequently, 2-bromo-naphthoquinone (100 mg, 0.42 mmol) dissolved in DMF (2 mL) was added to the reaction mixture, and the resulting solution was stirred at room temperature for 3 h under nitrogen. Afterwards, the solution was poured over ice water, which resulted in the formation of a precipitate. The precipitate was collected by vacuum filtration and washing of the filtrate with cold water, affording **3** as a tan solid (104.1 mg, 74% yield). The solid can be recrystallized from DCM and petroleum ether if needed. HPLC analysis: 98.2% AUC at 11.291 min, mp 156–157 °C. ^1^H NMR (500 MHz, CDCl_3_) δ 8.23–8.17 (m, 1H), 8.11–8.04 (m, 1H), 7.78 (dq, *J* = 7.6, 5.4, 3.8 Hz, 2H), 7.33 (d, *J* = 8.5 Hz, 2H), 7.22–7.15 (m, 2H), 5.98 (s, 1H). ^13^C NMR (126 MHz, CDCl_3_) δ 184.7, 179.6, 160.1, 150.9, 147.1, 147.1, 134.6, 133.7, 131.9, 131.0, 126.9, 126.3, 123.1, 122.5, 120.4 (q, J_CF_ = 258 Hz), 113.7, 77.3, 77.0, 76.8. HRMS (ESI): *m*/*z* calculated C_17_H_10_F_3_O_4_^+^ for [M+H]^+^: 335.0579, found 335.0536.

#### 4.1.4. N-(4-((1,4-Dioxo-1,4-dihydronaphthalen-2-yl)oxy)phenyl)acetamide (**4**)

The title compound **4** was prepared according to the method of Prati [19], and spectra were consistent with reported values.

#### 4.1.5. 2-(3,5-Dimethylphenoxy)naphthalene-1,4-dione (**5**)

A solution of 3,5-dimethylphenol (0.42 mmol) and K_2_CO_3_ (1.27 mmol) in anhydrous DMF (8.8 mL) was stirred at room temperature for 1 h under nitrogen. Subsequently, 2-bromonaphthoquinone (100 mg, 0.42 mmol) dissolved in DMF (2 mL) was added to the reaction mixture, and the resulting solution was stirred at room temperature for 3 h under nitrogen. Afterwards, the solution was poured over ice water and placed in the freezer, which resulted in the formation of a precipitate. The precipitate was collected by vacuum filtration, and washing of the filtrate with cold water resulted in a crude brown solid. The solid was then purified by flash column chromatography (1:9 EtOAc–hexane), which provided **5** as a light tan solid (62.4 mg, 53% yield). HPLC analysis: 99.4% AUC at 11.072 min, mp 130–132 °C. ^1^H NMR (500 MHz, CDCl_3_) δ 8.24–8.16 (m, 1H), 8.10–8.03 (m, 1H), 7.81–7.71 (m, 3H), 6.93 (d, *J* = 1.7 Hz, 1H), 6.76–6.72 (m, 2H), 5.99 (s, 1H), 2.34 (s, 6H). ^13^C NMR (126 MHz, CDCl_3_) δ 185.2, 180.1, 160.7, 152.6, 140.4, 134.4, 133.5, 132.0, 131.1, 128.2, 126.8, 126.2, 118.5, 113.3, 77.3, 77.0, 76.8, 21.3. HRMS (ESI): *m*/*z* calculated C_18_H_15_O_3_^+^ for [M+H]^+^: 279.0979, found 279.1016.

#### 4.1.6. 2-(Phenylamino)naphthalene-1,4-dione (**6**)

The title compound **6** was prepared according to the method of Prati [19], and spectra were consistent with reported values.

#### 4.1.7. 2-(Benzylamino)naphthalene-1,4-dione (**7**)

The title compound **7** was prepared according to the method of Prati [19], and spectra were consistent with reported values.

#### 4.1.8. 2-Methyl-3-(4-(trifluoromethoxy)phenoxy)naphthalene-1,4-dione (**8**)

2-(4-(trifluoromethoxy)phenoxy)naphthalene-1,4-dione **3** (0.71 mmol), AgNO_3_ (0.36 mmol), glacial acetic acid (0.71 mmol), and ACN (3.4 mL) were added to a vial, and the solution was stirred at room temperature for 5 min. Afterwards, a separate solution of ammonium persulfate (1.28 mmol) in water (0.92 mL H_2_O) was added to the reaction mixture dropwise, and the resulting solution was set to stir at 60 °C for 1 h under nitrogen. A second portion of glacial acetic acid was added (0.36 mmol), followed by a second solution of ammonium persulfate (1.28 mmol) in water (0.92 mL H_2_O), and the resulting solution was stirred at 70 °C for 5 h under nitrogen. The reaction was allowed to cool to room temperature and then vacuum-filtered. The filtrate was collected, diluted with water, and extracted with EtOAc (3×). The extractions were combined, washed with brine (2×), and dried over anhydrous Na_2_SO_4_. The solvent was removed in vacuo and the resulting crude product was purified by column chromatography (neat hexane) to provide **8** as bright yellow crystals (90.8 mg, 37% yield). Product can be recrystallized from neat hexane if needed. HPLC analysis: 96.1% AUC at 11.889 min, mp 102–103 °C. ^1^H NMR (500 MHz, CDCl_3_) δ 8.18–8.13 (m, 1H), 8.06–8.01 (m, 1H), 7.75 (dtd, *J* = 17.6, 7.4, 1.5 Hz, 2H), 7.17 (dtd, *J* = 8.7, 2.4, 1.4 Hz, 2H), 7.00–6.93 (m, 2H), 2.18 (s, 3H). ^13^C NMR (126 MHz, CDCl_3_) δ 185.3, 179.3, 155.4, 153.3, 144.5, 136.3, 134.2, 133.9, 132.1, 131.1, 126.6, 122.7, 122.5, 120.4 (q, J_CF_ = 256 Hz), 116.9, 9.9. HRMS (ESI): *m*/*z* calculated C_18_H_12_F_3_O_4_^+^ for [M+H]^+^: 349.0679, found 349.0682.

#### 4.1.9. 2-(4-Fluorophenoxy)-3-methylnaphthalene-1,4-dione (**9**)

2-(4-fluorophenoxy)naphthalene-1,4-dione **1** (0.73 mmol), AgNO_3_ (0.37 mmol), glacial acetic acid (0.73 mmol), and ACN (3.5 mL) were added to a vial, and the solution was stirred at room temperature for 5 min. Afterwards, a separate solution of ammonium persulfate (1.32 mmol) in water (0.95 mL H_2_O) was added to the reaction mixture dropwise, and the resulting solution was set to stir at 60 °C for 1 h under nitrogen. A second portion of glacial acetic acid was added (0.37 mmol), followed by a second solution of ammonium persulfate (1.32 mmol) in water (0.95 mL H_2_O), and the resulting solution was stirred at 70 °C for 5 h under nitrogen. The reaction was allowed to cool to room temperature and then vacuum-filtered. The filtrate was collected, diluted with water, and extracted with DCM (4×). The extractions were combined and dried over anhydrous Na_2_SO_4_, and the solvent was evaporated under reduced pressure, resulting in a crude product. The solid was purified by column chromatography using a step gradient of neat hexane, followed by 5:95 EtOAc–hexane, affording a crude solid. The solid was then recrystallized from neat hexane, affording the title compound as bright yellow crystals (11.5 mg, 11% yield). Product can be recrystallized from neat hexane if necessary. HPLC analysis: 98.7% pure AUC at 11.001 min, mp 122–123 °C. ^1^H NMR (500 MHz, CDCl_3_) δ 8.17–8.12 (m, 1H), 8.05–7.99 (m, 1H), 7.79–7.68 (m, 2H), 7.04–6.96 (m, 2H), 6.96–6.88 (m, 2H), 2.18 (s, 3H). ^13^C NMR (126 MHz, CDCl_3_) δ 185.4, 179.5, 158.6 (d, J_CF_ = 242 Hz), 153.7, 153.1 (d, J_CF_ = 1.3 Hz), 135.8, 134.1, 133.8, 132.1, 131.1, 126.6, 117.2 (d, J_CF_ = 24 Hz), 116.4 (d, J_CF_ = 24 Hz), 77.3, 77.0, 76.8, 9.8. HRMS (ESI): *m*/*z* calculated C_17_H_12_FO_3_^+^ for [M+H]^+^: 283.0779, found 283.0765.

#### 4.1.10. 2-Methyl-3-(phenylamino)naphthalene-1,4-dione (**11**)

The title compound **11** was prepared according to the method of Smarma [27], and spectra were consistent with reported values.

#### 4.1.11. 2-((2-(4-(Trifluoromethoxy)phenoxy)phenyl)amino)naphthalene-1,4-dione (**12**)

Pd(OAc)_2_ (5 mol %), racemic-BINAP (10 mol %), amine (0.18 mmol), K_2_CO_3_ (0.30 mmol), 2-bromo-naphthoquinone (36 mg, 0.15 mmol), and toluene (0.9 mL) were added to a vial, and the vial was backfilled with argon. The resulting solution was stirred at 90 °C for 24 h. Afterwards, the solution was allowed to cool to room temperature and the toluene was evaporated under reduced pressure. The blackish residue was then quenched with acetone and filtered through a bed of celite. The acetone was then stripped off in vacuo, and the residue was purified by column chromatography (1:9 EtOAc–hexane), affording **12** as red crystals (56.6 mg, 87.5% yield). HPLC analysis: 99.5% pure AUC at 12.286 min, mp 110–111 °C. ^1^H NMR (500 MHz, CDCl_3_) δ 8.03 (ddd, *J* = 10.2, 7.7, 1.4 Hz, 2H), 7.76 (s, 1H), 7.69 (td, *J* = 7.6, 1.4 Hz, 1H), 7.59 (td, *J* = 7.5, 1.3 Hz, 1H), 7.47 (dd, *J* = 8.0, 1.6 Hz, 1H), 7.15 (td, *J* = 7.7, 1.5 Hz, 1H), 7.15–7.09 (m, 2H), 7.12–7.06 (m, 1H), 6.99–6.92 (m, 2H), 6.93 (dd, *J* = 8.1, 1.5 Hz, 1H), 6.41 (s, 1H). ^13^C NMR (126 MHz, CDCl_3_) δ 184.0, 181.8, 154.8, 148.7, 145.0, 145.0, 144.0, 134.9, 133.1, 132.5, 130.4, 129.3, 126.6, 126.2, 126.0, 124.6, 122.8, 122.7, 121.5, 119.4, 119.4, 104.2, 77.3, 77.0, 76.8. HRMS (ESI): *m*/*z* calculated C_23_H_15_F_3_NO_4_^+^ for [M+H]^+^: 426.0979, found 426.0948.

#### 4.1.12. N-(4-(2-((1,4-Dioxo-1,4-dihydronaphthalen-2-yl)amino)phenoxy)phenyl)acetamide (**13**)

Pd(OAc)_2_ (5 mol %), racemic-BINAP (10 mol %), amine (0.33 mmol), K_2_CO_3_ (0.55 mmol), 2-bromo-naphthoquinone (65 mg, 0.27 mmol), and toluene (1.6 mL) were added to a vial, and the vial was backfilled with argon. The resulting solution was stirred at 90 °C for 16 h. Afterwards, the solution was allowed to cool to room temperature, and the toluene was evaporated under reduced pressure. The blackish residue was then quenched with acetone and filtered through a bed of celite. The acetone was then stripped off in vacuo, and the residue was purified by column chromatography (1:1 EtOAc–hexane). The collected solid was then recrystallized from methanol to provide the title compound as shiny red prismatic crystals (65.3 mg, 60% yield). HPLC analysis: 98.4% pure AUC at 10.461 min, mp 214–215 °C. ^1^H NMR (500 MHz, CDCl_3_) δ 8.10 (ddd, *J* = 8.2, 7.1, 1.3 Hz, 2H), 7.92 (s, 1H), 7.75 (td, *J* = 7.5, 1.4 Hz, 1H), 7.66 (td, *J* = 7.6, 1.4 Hz, 1H), 7.54–7.44 (m, 3H), 7.27 (s, 1H), 7.16 (td, *J* = 7.7, 1.5 Hz, 1H), 7.11 (td, *J* = 7.8, 1.7 Hz, 1H), 7.05–6.96 (m, 2H), 6.93 (dd, *J* = 8.0, 1.5 Hz, 1H), 6.50 (s, 1H), 2.17 (s, 3H). ^13^C NMR (126 MHz, CDCl_3_) δ 184.0, 181.8, 168.2, 152.4, 149.4, 144.1, 134.8, 134.1, 133.2, 132.4, 130.4, 128.8, 126.6, 126.1, 125.8, 123.8, 122.3, 121.7, 119.4, 118.5, 104.0, 77.3, 77.0, 76.8, 24.5. HRMS (ESI): *m*/*z* calculated C_24_H_19_N_2_O_4_^+^ for [M+H]^+^: 399.1379, found 399.1339.

#### 4.1.13. 2-Methyl-3-((2-(4-(trifluoromethoxy)phenoxy)phenyl)amino)naphthalene-1,4-dione (**14**)

Pd(OAc)_2_ (5 mol %), racemic-BINAP (10 mol %), amine (0.24 mmol), K_2_CO_3_ (0.40 mmol), 2-bromo-3-methylnaphthalene-1,4-dione **10** (50 mg, 0.20 mmol), and toluene (1.2 mL) were added to a vial, and the solution was backfilled with argon. The resulting reaction mixture was stirred at 90 °C for 16 h. Afterwards, the solution was allowed to cool to room temperature, and the toluene was evaporated under reduced pressure. The blackish residue was then quenched with warm ACN and filtered through a bed of celite. The solvent was then stripped off in vacuo, and the residue was purified by column chromatography (1:9 EtOAc–hexane). The collected solid was then recrystallized from neat hexane to provide **14** as fluffy red crystals (10.6 mg, 12% yield). HPLC analysis: 97.5% pure AUC at 12.595 min, mp 140–142 °C. ^1^H NMR (500 MHz, CDCl_3_) δ 8.05 (dd, *J* = 7.6, 1.3 Hz, 1H), 7.96 (dd, *J* = 7.6, 1.5 Hz, 1H), 7.65 (td, *J* = 7.5, 1.4 Hz, 1H), 7.57 (td, *J* = 7.5, 1.4 Hz, 1H), 7.12–7.04 (m, 3H), 7.01 (td, *J* = 7.8, 1.7 Hz, 1H), 6.93 (dd, *J* = 8.1, 1.5 Hz, 1H), 6.91–6.82 (m, 3H), 1.75 (s, 3H). ^13^C NMR (126 MHz, CDCl_3_) δ 184.4, 182.1, 155.3, 147.9, 144.5, 142.1, 134.4, 133.1, 132.6, 131.8, 130.4, 126.4, 126.2, 124.6, 124.3, 122.8, 121.5, 120.4, 120.2, 119.9 (q, J_CF_ = 241 Hz), 118.5, 13.7. HRMS (ESI): *m*/*z* calculated C_24_H_17_F_3_NO_4_^+^ for [M+H]^+^: 440.1079, found 440.1104.

#### 4.1.14. N-(4-(2-((3-Methyl-1,4-dioxo-1,4-dihydronaphthalen-2-yl)amino)phenoxy)phenyl)acetamide (**15**)

Pd(OAc)_2_ (5 mol %), racemic-BINAP (10 mol %), amine (0.31 mmol), K_2_CO_3_ (0.52 mmol), 2-bromo-3-methylnaphthalene-1,4-dione **10** (65 mg, 0.26 mmol), and toluene (1.5 mL) were added to a vial, and the solution was backfilled with argon. The resulting solution was stirred at 90 °C for 24 h. Afterwards, the solution was allowed to cool to room temperature, and the toluene was evaporated under reduced pressure. The blackish residue was then quenched with DCM and filtered through a bed of celite. The solvent was then stripped off in vacuo, and the black–red thick oily residue was purified by column chromatography (7:3 EtOAc–hexane). The isolated crude solid was then dissolved in EtOAc and washed with 1.0 M HCl (3×), saturated NaHCO_3_ (2×), water (2×), and brine (2×) and dried over anhydrous Na_2_SO_4_. The solvent was evaporated under reduced pressure, affording *N*-(4-(2-((3-methyl-1,4-dioxo-1,4-dihydronaphthalen-2-yl)amino)phenoxy)phenyl)acetamide **15** as a dark red solid (18 mg, 17% yield). HPLC analysis: 98.6% pure AUC at 10.801 min, mp 206–207 °C. ^1^H NMR (500 MHz, CDCl_3_) δ 8.04 (dd, *J* = 7.6, 1.3 Hz, 1H), 7.97 (dd, *J* = 7.7, 1.3 Hz, 1H), 7.64 (td, *J* = 7.5, 1.4 Hz, 1H), 7.56 (td, *J* = 7.6, 1.4 Hz, 1H), 7.40–7.33 (m, 2H), 7.25 (s, 1H), 7.11 (s, 1H), 7.02 (td, *J* = 7.6, 1.5 Hz, 1H), 6.97 (td, *J* = 7.7, 1.7 Hz, 1H), 6.90–6.83 (m, 3H), 6.80 (dd, *J* = 7.9, 1.7 Hz, 1H), 2.10 (s, 3H), 1.76 (s, 3H). ^13^C NMR (126 MHz, CDCl_3_) δ 184.5, 182.2, 168.1, 153.1, 148.7, 142.2, 134.3, 133.5, 133.2, 132.5, 131.3, 130.5, 126.3, 126.2, 124.4, 123.4, 122.6, 121.7, 120.0, 119.2, 118.6, 77.3, 77.0, 76.8, 24.5, 13.8. HRMS (ESI): *m*/*z* calculated C_25_H_21_N_2_O_4_^+^ for [M+H]^+^: 413.1479, found 413.1496.

#### 4.1.15. 2-((4-(4-(Trifluoromethoxy)phenoxy)phenyl)amino)naphthalene-1,4-dione (**16**)

Pd(OAc)_2_ (5 mol %), racemic-BINAP (10 mol %), amine (0.28 mmol), K_2_CO_3_ (0.47 mmol), 2-hydroxy-3-iodonaphthalene-1,4-dione (70 mg, 0.26 mmol), and toluene (1.5 mL) were added to a vial, and the solution was backfilled with argon. The resulting solution was stirred at 90 °C for 19 h. Afterwards, the solution was allowed to cool to room temperature and filtered over a bed of celite. The resulting red filtrate was collected and concentrated in vacuo. This residue was then quenched with 1.0 M HCl and then extracted with EtOAc (4×). The organic extracts were combined and washed with 1.0 M HCl (2×), saturated NaHCO_3_ (2×), water (2×), and brine (2×) and dried over anhydrous Na_2_SO_4_, resulting in a crude red solid. The solid was subject to column chromatography using a step gradient of neat hexane, followed by 1:9 EtOAc–hexane to afford a red solid crude material, which was further purified by recrystallizing from neat hexane to afford the title compound as a dark red solid (23.7 mg, 23% yield). HPLC analysis: 96.2% pure AUC at 12.552 min, mp 166–168 °C. ^1^H NMR (500 MHz, CDCl_3_) δ 8.12 (td, *J* = 8.1, 1.3 Hz, 2H), 7.77 (td, *J* = 7.5, 1.4 Hz, 1H), 7.68 (td, *J* = 7.5, 1.4 Hz, 1H), 7.49 (s, 1H), 7.30–7.18 (m, 4H), 7.11–7.00 (m, 4H), 6.31 (s, 1H). ^13^C NMR (126 MHz, CDCl_3_) δ 183.8, 182.0, 155.5, 154.6, 145.2, 144.8, 135.0, 133.3, 133.0, 132.4, 130.4, 126.6, 126.2, 124.8, 122.8, 120.5 (q, J_CF_ = 257 Hz), 120.1, 119.7, 103.1. HRMS (ESI): *m*/*z* calculated C_23_H_15_F_3_NO_4_^+^ for [M+H]^+^: 426.0979 found 426.0956.

### 4.2. NQR Purification

*V. cholerae* NQR was purified using the methods described by our group [28]. NQR-expressing cells were harvested, washed by centrifugation, and lysed via high-pressure homogenizer. The NQR complex was purified using Ni-NTA and DEAE ion-exchange chromatography. Protein purity (>95%) was verified by 30% urea and 15% SDS-PAGE acrylamide gel analysis.

### 4.3. Activity Measurements

NQR activity was measured spectrophotometrically following the reduction of ubiquinone at 282 nm and NADH at 340 nm [7,28,29] using UQ-1, NaCl, and NADH as substrates. The activity measurements were performed in TEG buffer solution (50 mM NaCl, 50 mM Tris, 1 mM EDTA, 5% glycerol, and 0.05% n-dodecyl-β-D-maltoside, pH of 8.0). The reaction mixture contained 2.5 nM NQR, 250 μM NADH, 50 μM UQ-1, and different concentrations of the inhibitors. All experiments were performed in quadruplicate.

### 4.4. Cytotoxicity Measurements

Cytotoxicity of the compounds was measured in triplicate using HeLa cells grown in Eagle’s Minimum Essential Medium supplemented with 10% (*v*/*v*) fetal bovine serum and penicillin/streptomycin (100 units/mL and 100 µg/mL, respectively), and HFF-1 cells grown in Dulbecco’s Modified Eagle Medium enriched with 15% fetal bovine serum and antibiotics, as described for HeLa cells. Cells were incubated at 37 °C with 5% CO_2_ in a humidified atmosphere and cultured in 96-well plates at a density of 8000 cells/well, followed by an incubation of one doubling time. Antibiotics were washed out, and the different compounds were added at a concentration of 10 µM and incubated for 24 h. Cell viability was assessed using Alamar Blue™ (Thermo Fisher Scientific, Waltham, MA, USA) as follows: Cells were incubated with the vital dye at 37 °C for 4 h, and then fluorescence was measured with excitation and emission wavelengths set at 570 nm and at 600 nm. Negative controls consisted of cells exposed solely to solvent (DMSO or ethanol).

### 4.5. Docking

The compounds evaluated in this work were docked to the cryo-EM structure of *Vibrio cholerae* NQR obtained from the RCSB Protein Data Bank (PDBID: 8EVU), parametrized with the AMBER ff14SB force field [30]. This structure is similar to other reported NQR structures [23,24]. The 3D structures of napthoquinones were generated using the SMILES translator in UCSF Chimera 1.16 [31], and subsequently minimized using AMBER ff14SB [30], hydrogenated, and parameterized using Gasteiger partial charges. Structures were docked to the ubiquinone binding site using Autodock Vina (version 1.2.0) [32,33].

## Figures and Tables

**Table 1:** Structure activity relationships (SAR) of the 1,4-naphthoquinone series.

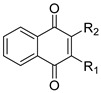							
Cmpd	R_1_	R_2_	cLogP	NQR % Inhibition		% Toxicity at 10 μM	
				10 μM	50 μM	HeLa	HFF-1
1,4-naphthoquinone	H	H	1.5	56 ± 6	100 ± 2	100 ± 6	100 ± 7
**1**	H	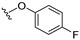	4.32	27 ± 2	57 ± 4	8 ± 6	0 ± 14
**2**	H	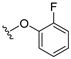	4.12	33 ± 4	62 ± 2	100 ± 6	100 ± 6
**3**	H	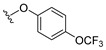	5.23	31 ± 2	79 ± 1	33 ± 9	0 ± 53
**4**	H	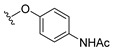	3.21	29 ± 3.0	70 ± 9	47 ± 26	0 ± 68
**5**	H	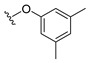	5.03	32 ± 1	67 ± 1	100 ± 12	30 ± 44
**6**	H	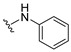	3.98	13 ± 1	42.7 ± 1	22 ± 16	25 ± 12
**7**	H	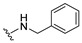	3.80	32 ± 2	44 ± 1	19 ± 12	0 ± 8
**8**	CH_3_	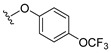	5.75	21 ± 5	58 ± 7	45 ± 24	9 ± 10
**9**	CH_3_	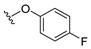	4.84	22 ± 8	46 ± 1	17 ± 10	0 ± 20
**11**	CH_3_	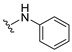	4.49	33 ± 7	63 ± 1	23 ± 11	0 ± 9
**12**	H	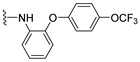	7.1	76 ± 3	96 ± 2	5 ± 11	0 ± 24
**13**	H	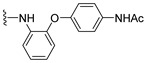	5.09	30 ± 6	68 ± 8	16 ± 6	0 ± 9
**14**	CH_3_	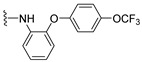	7.62	72 ± 8	93 ± 3	5 ± 8	10 ± 7
**15**	CH_3_	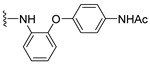	5.61	51 ± 2	99 ± 3	18 ± 6	0 ± 8
**16**	H	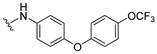	7.1	24 ± 14	74 ± 4	19 ± 9	0 ± 10

**Table 2:** IC_50_ values of select naphthoquinones.

Compound	IC_50_ (μM)
HQNO	0.2
1,4-Naphthoquinone	9 ± 3
**12**	12 ± 1
**14**	16 ± 3
**16**	46 ± 5
**6**	>100

## Data Availability

The original contributions presented in this study are included in the article/Supplementary Materials. Further inquiries can be directed to the corresponding author(s).

## Supplementary Materials

The following supporting information can be downloaded at: https://www.mdpi.com/article/10.3390/ijms27031198/s1.
