# Supplementary material for: Synthesis, Biological Evaluation, and Computational Analysis of 1,4-Naphthoquinone Derivatives as Inhibitors of the Sodium-Dependent NADH:Ubiquinone Oxidoreductase (NQR) in Vibrio cholerae
Source: Int J Mol Sci. 2026 Jan 24;27(3):1198. doi: 10.3390/ijms27031198 (PMC12898190; doi:10.3390/ijms27031198)

Supporting Information

Synthesis, Biological Evaluation, and Computational Analysis of 1,4-Naphthoquinone Derivatives as Inhibitors of the Sodium-Dependent NADH:Ubiquinone Oxidoreductase (NQR) in Vibrio cholerae

Zachary Liveris[1], Ming Yuan[2], Karina Tuz[2], Oscar Juarez[2*], and Daniel P. Becker[1*]

[1]Department of Chemistry and Biochemistry, Loyola University Chicago, 1032 West Sheridan Road, Chicago, IL 60660

[2]Department of Biological Sciences, Illinois Institute of Technology, Chicago, IL, 60616

Table of Contents

Pages	Compound
2-5	2-(2-fluorophenoxy)naphthalene-1,4-dione (**2, 1-27**)
6-9	2-(4-(trifluoromethoxy)phenoxy)naphthalene-1,4-dione (**3, 1-25**)
10-13	2-(3,5-dimethylphenoxy)naphthalene-1,4-dione (**5, 1-24**)
14-17	2-methyl-3-(4-(trifluoromethoxy)phenoxy)naphthalene-1,4-dione (**8, 1-20**)
18-21	2-(4-fluorophenoxy)-3-methylnaphthalene-1,4-dione (**9, 1-22**)
22-25	2-((2-(4-(trifluoromethoxy)phenoxy)phenyl)amino)naphthalene-1,4-dione (**12, 1-21**)
26-29	N-(4-(2-((1,4-dioxo-1,4-dihydronaphthalen-2-yl)amino)phenoxy)phenyl)acetamide (**13, 1-16**)
30-33	2-methyl-3-((2-(4-(trifluoromethoxy)phenoxy)phenyl)amino)naphthalene-1,4-dione (**14, 1-19**)
34-37	N-(4-(2-((3-methyl-1,4-dioxo-1,4-dihydronaphthalen-2-yl)amino)phenoxy)phenyl)acetamide (**15, 1-31**)
38-41	2-((4-(4-(trifluoromethoxy)phenoxy)phenyl)amino)naphthalene-1,4-dione (**16, 4-10**)

HPLC of 2-(2-fluorophenoxy)naphthalene-1,4-dione (**2, 1-27**)

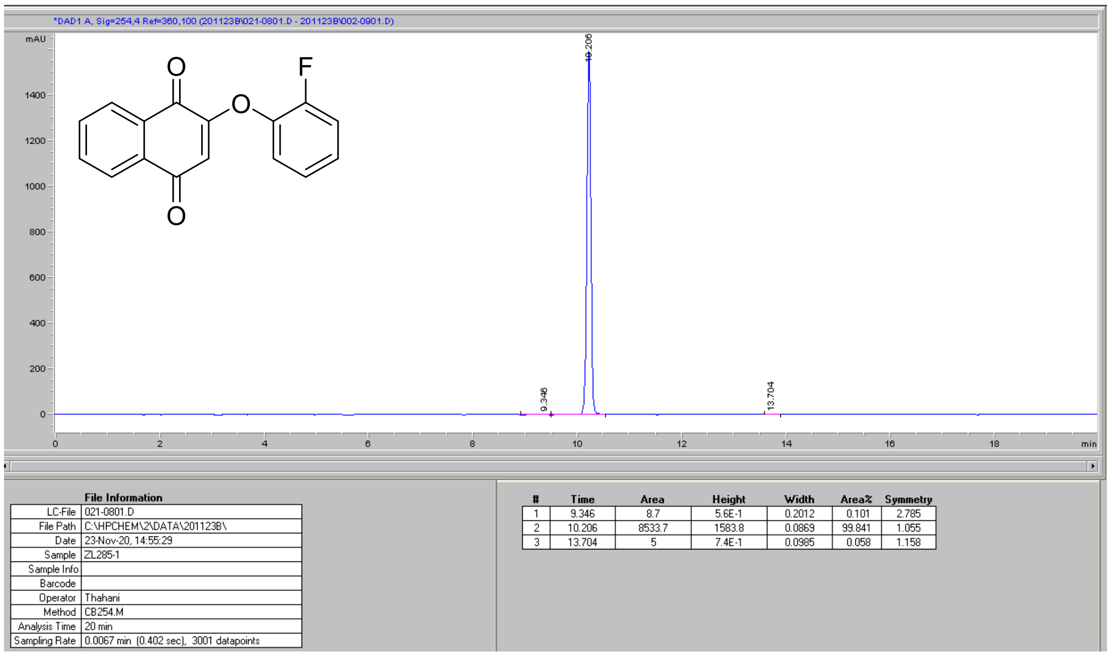

File Information

LC-File	021-0801.D
File Path	C:\HPCHEM\2\DATA\201123B\
Date	23-Nov-20, 14:55:29
Sample	ZL285-1
Sample Info	
Barcode	
Operator	Thahani
Method	CB254.M
Analysis Time	20 min
Sampling Rate	0.0067 min (0.402 sec), 3001 datapoints

#	Time	Area	Height	Width	Area%	Symmetry
1	9.346	8.7	5.6E-1	0.2012	0.101	2.785
2	10.206	8533.7	1583.8	0.0869	99.841	1.055
3	13.704	5	7.4E-1	0.0985	0.058	1.158

^{1}H NMR of 2-(2-fluorophenoxy)naphthalene-1,4-dione (**2, 1-27**)

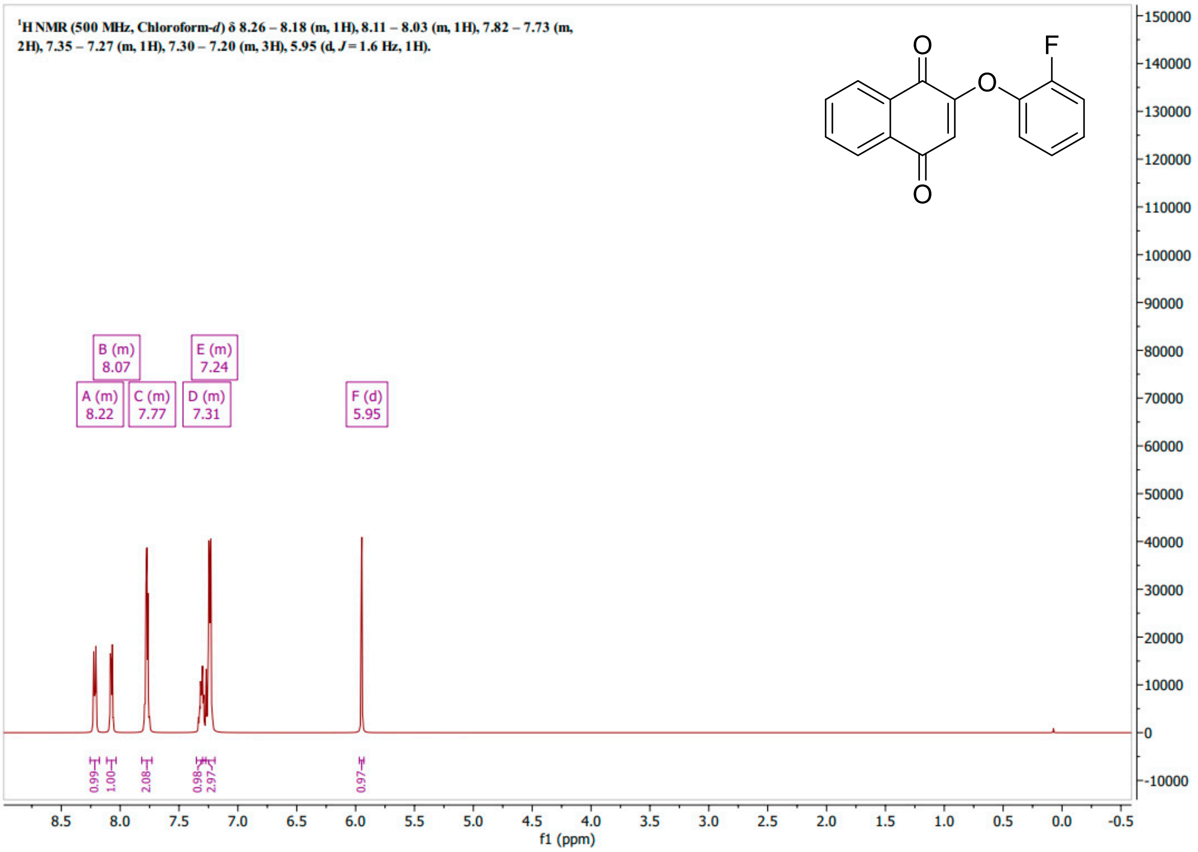

^{13}C NMR of 2-(2-fluorophenoxy)naphthalene-1,4-dione (**2, 1-27**)

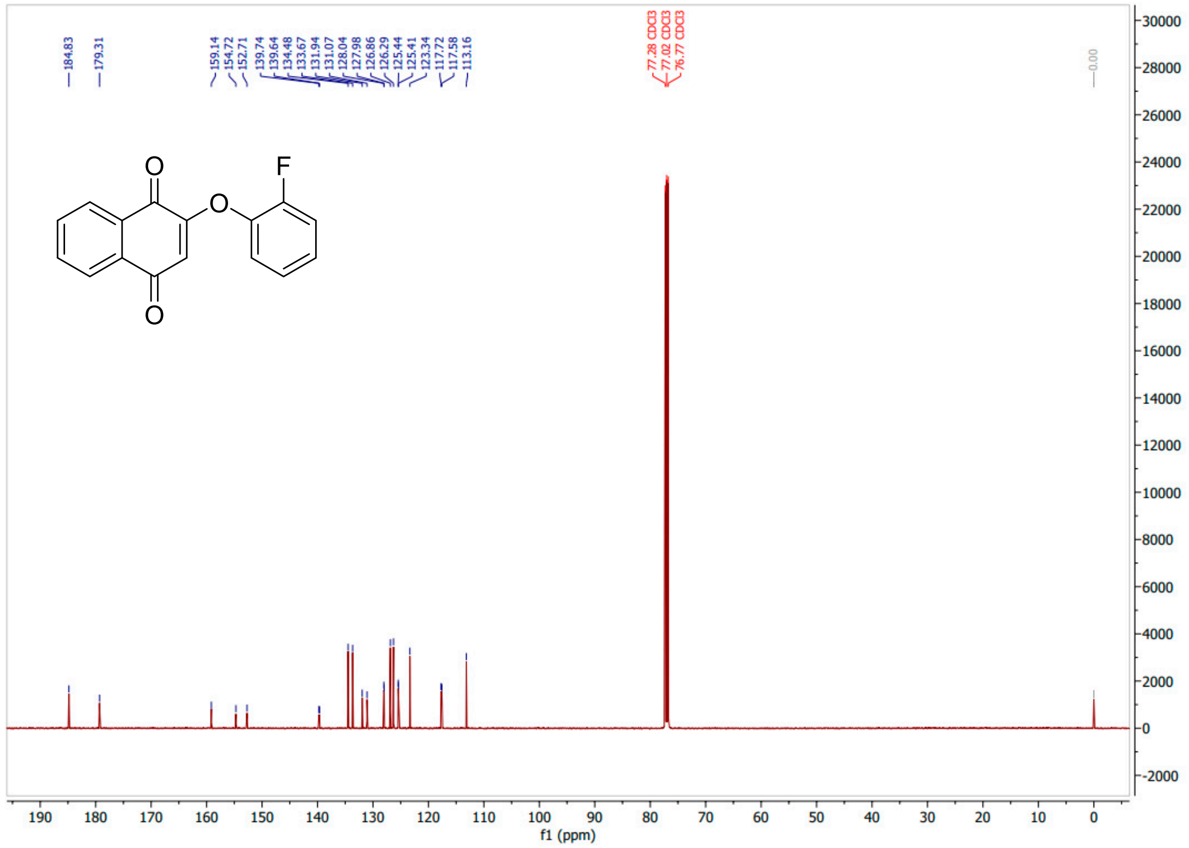

HRMS of 2-(2-fluorophenoxy)naphthalene-1,4-dione (**2, 1-27**)

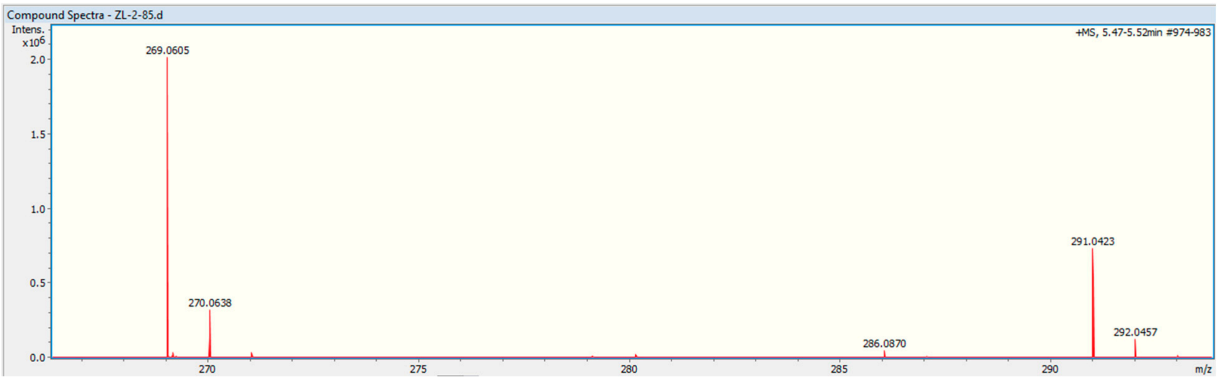

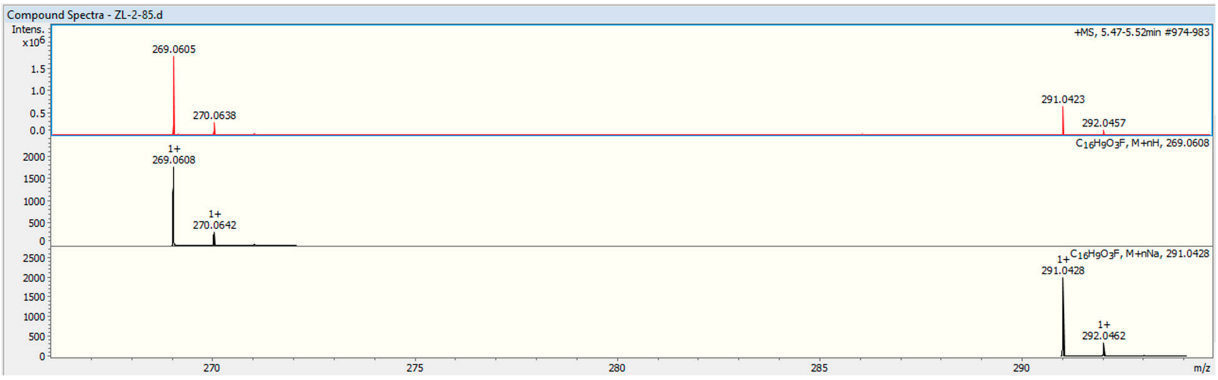

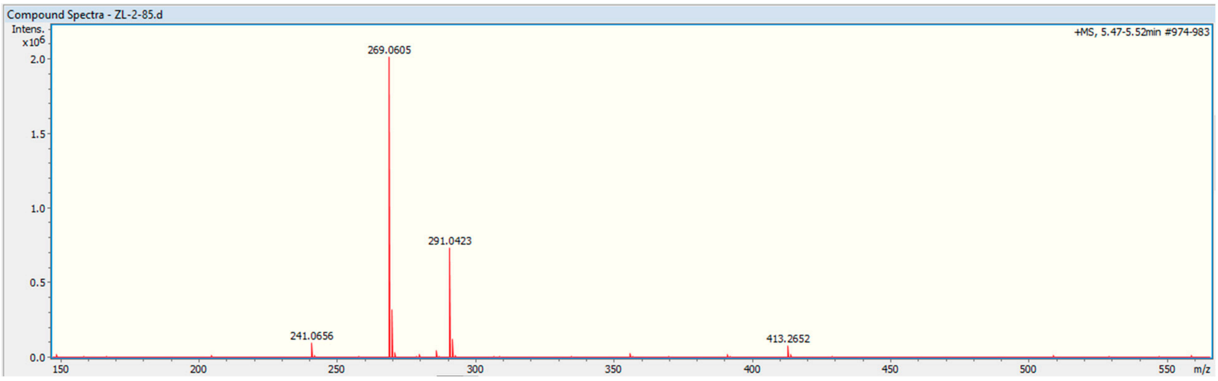

HPLC of 2-(4-(trifluoromethoxy)phenoxy)naphthalene-1,4-dione (**3, 1-25**)

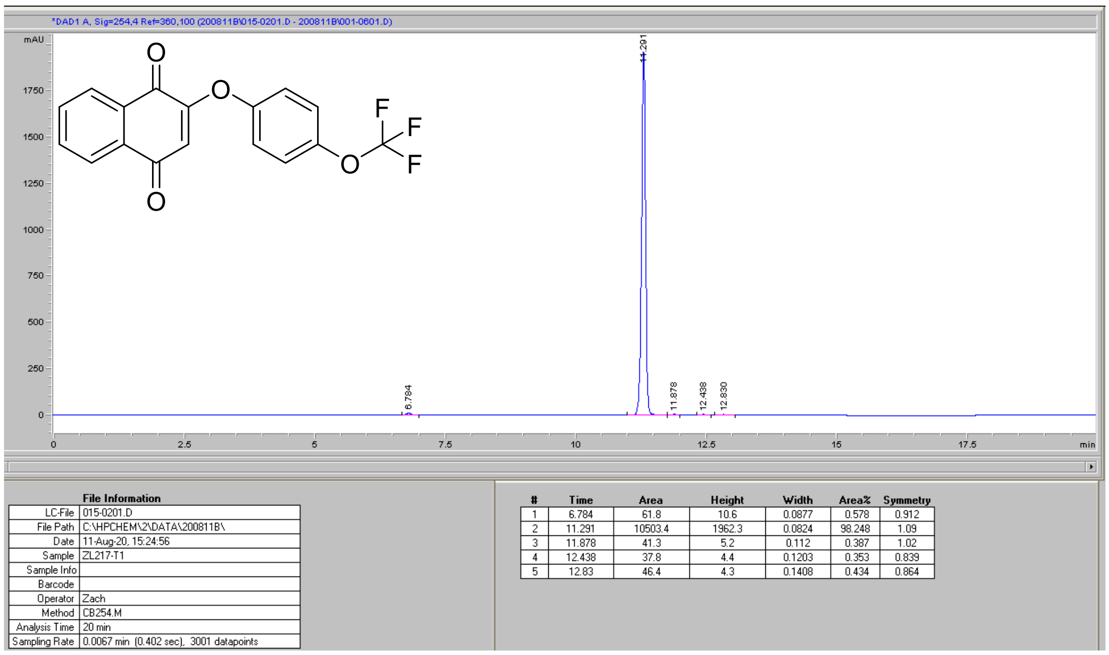

File Information

LC-File	015-0201.D
File Path	C:\HPCHEM\2\DATA\200811B\
Date	11-Aug-20, 15:24:56
Sample	ZL217-T1
Sample Info	
Barcode	
Operator	Zach
Method	CB254.M
Analysis Time	20 min
Sampling Rate	0.0067 min (0.402 sec), 3001 datapoints

#	Time	Area	Height	Width	Area%	Symmetry
1	6.784	61.8	10.6	0.0877	0.578	0.912
2	11.291	10503.4	1962.3	0.0824	98.248	1.09
3	11.878	41.3	5.2	0.112	0.387	1.02
4	12.438	37.8	4.4	0.1203	0.353	0.839
5	12.83	46.4	4.3	0.1408	0.434	0.864

^{1}H NMR of 2-(4-(trifluoromethoxy)phenoxy)naphthalene-1,4-dione (**3, 1-25**)

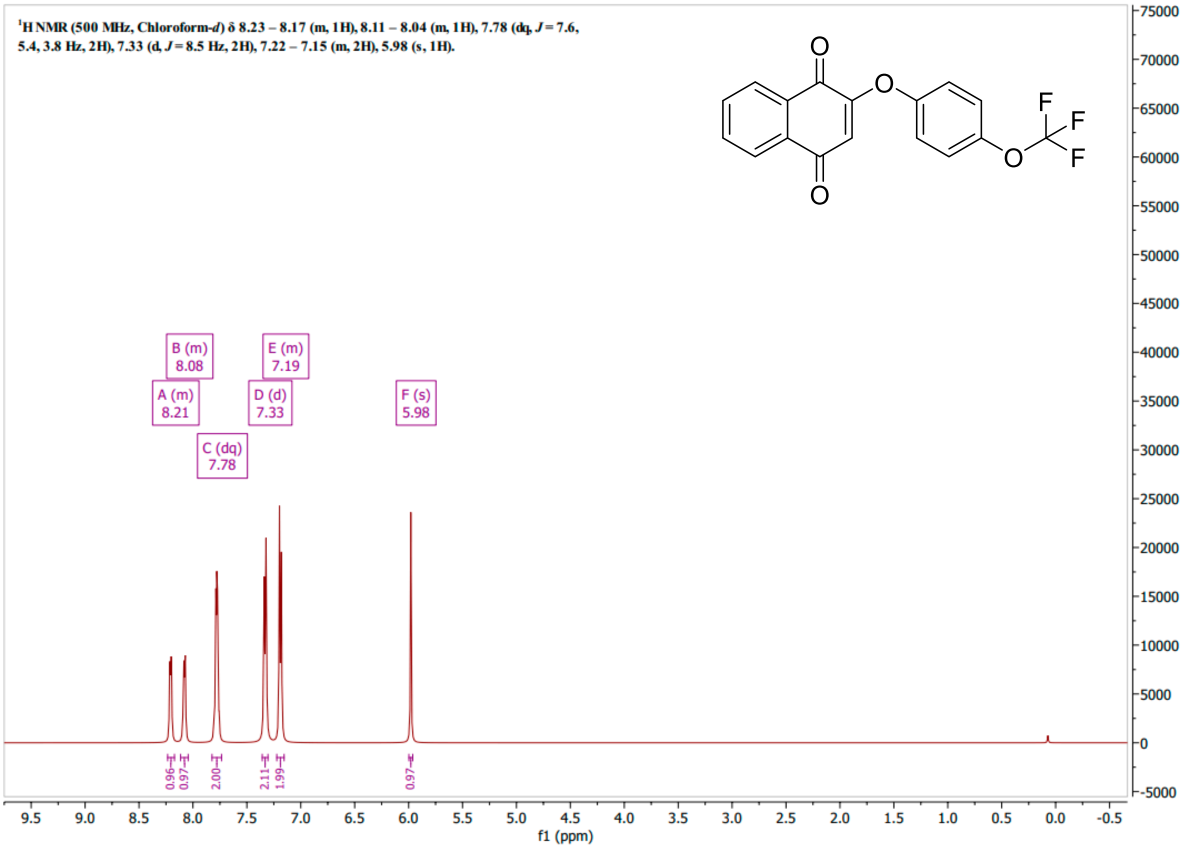

^{13}C NMR of 2-(4-(trifluoromethoxy)phenoxy)naphthalene-1,4-dione (**3, 1-25**)

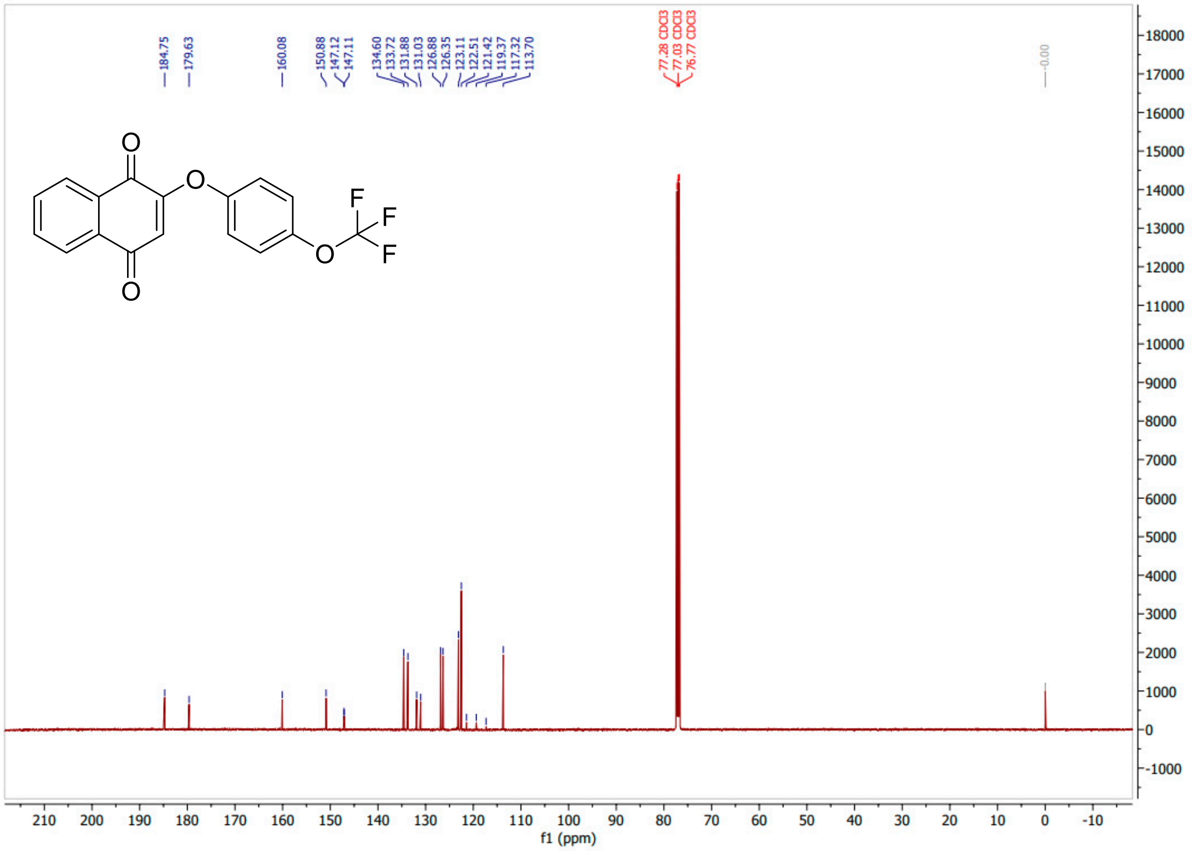

HRMS of 2-(4-(trifluoromethoxy)phenoxy)naphthalene-1,4-dione (**3, 1-25**)

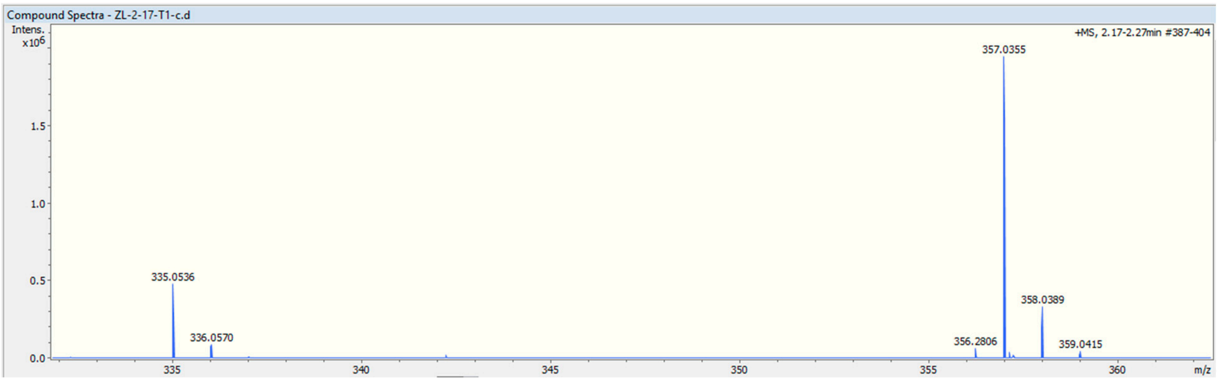

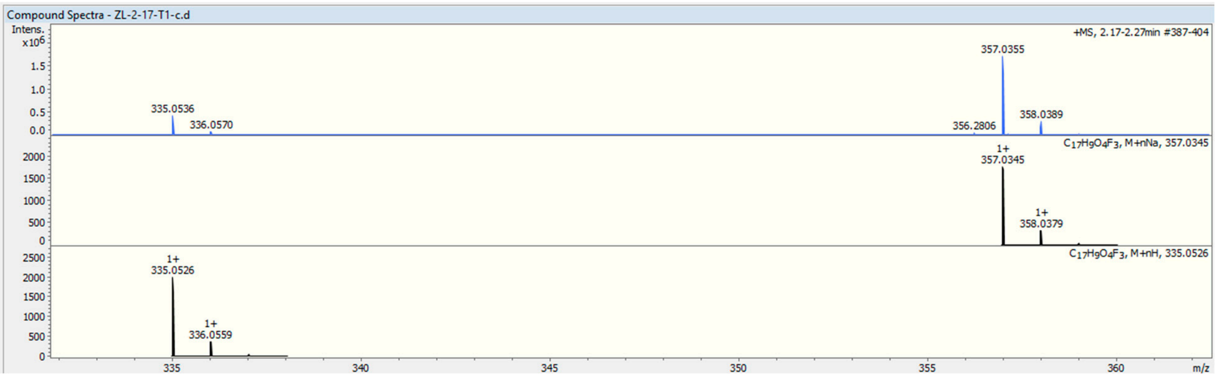

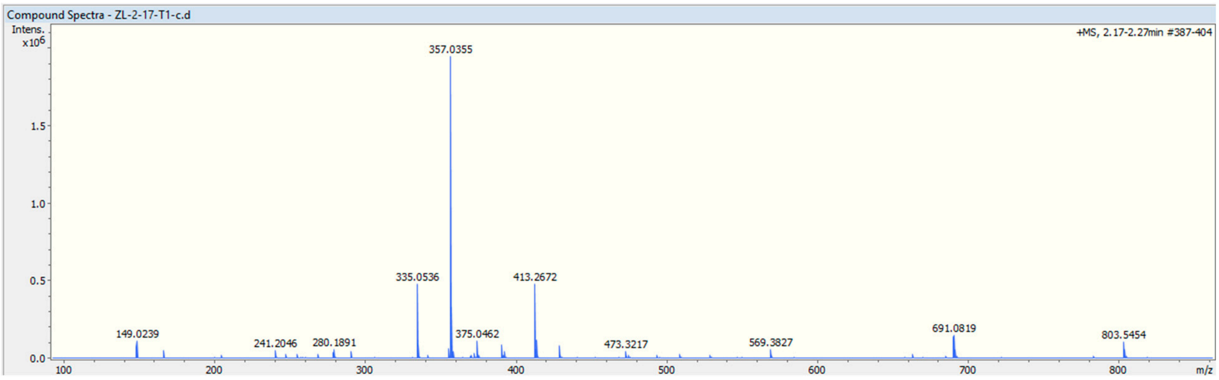

HPLC of 2-(3,5-dimethylphenoxy)naphthalene-1,4-dione (**5, 1-24**)

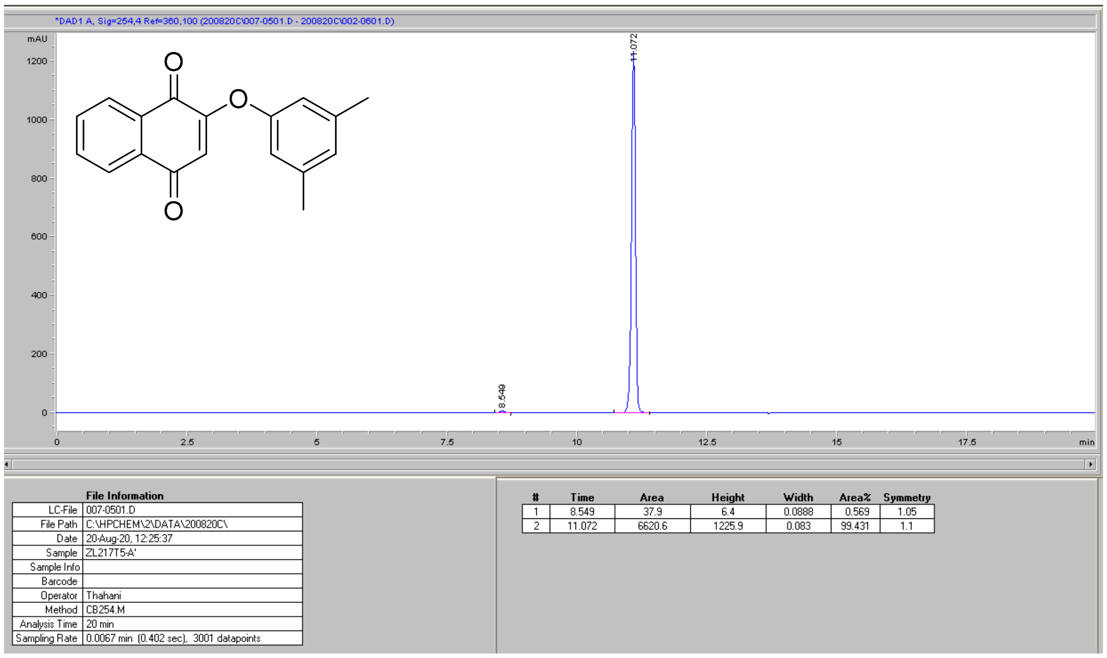

File Information

LC-File	007-0501.D
File Path	C:\HPCHEM\2\DATA\200820C\
Date	20-Aug-20, 12:25:37
Sample	ZL217T5-A'
Sample Info	
Barcode	
Operator	Thahani
Method	CB254.M
Analysis Time	20 min
Sampling Rate	0.0067 min (0.402 sec), 3001 datapoints

#	Time	Area	Height	Width	Area%	Symmetry
1	8.549	37.9	6.4	0.0888	0.569	1.05
2	11.072	6620.6	1225.9	0.083	99.431	1.1

^{1}H NMR of 2-(3,5-dimethylphenoxy)naphthalene-1,4-dione (**5, 1-24**)

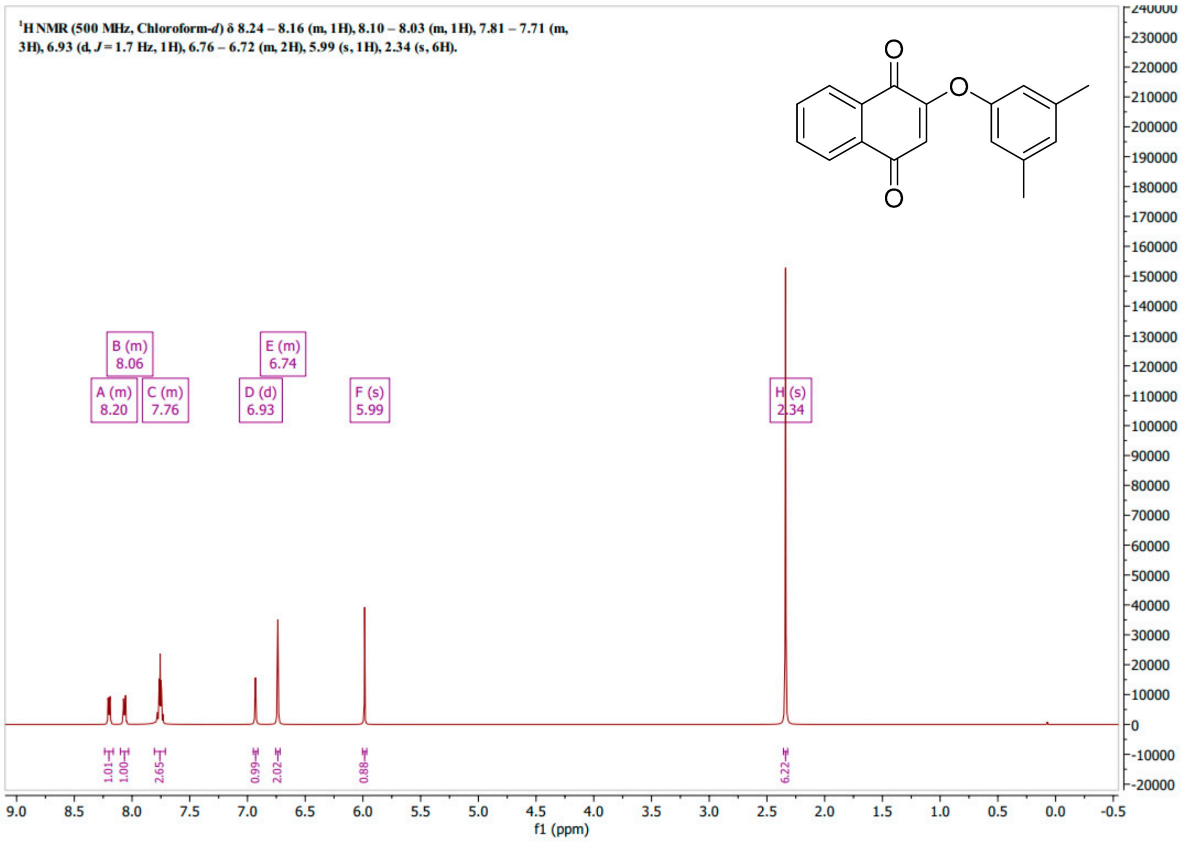

^{13}C NMR of 2-(3,5-dimethylphenoxy)naphthalene-1,4-dione (**5, 1-24**)

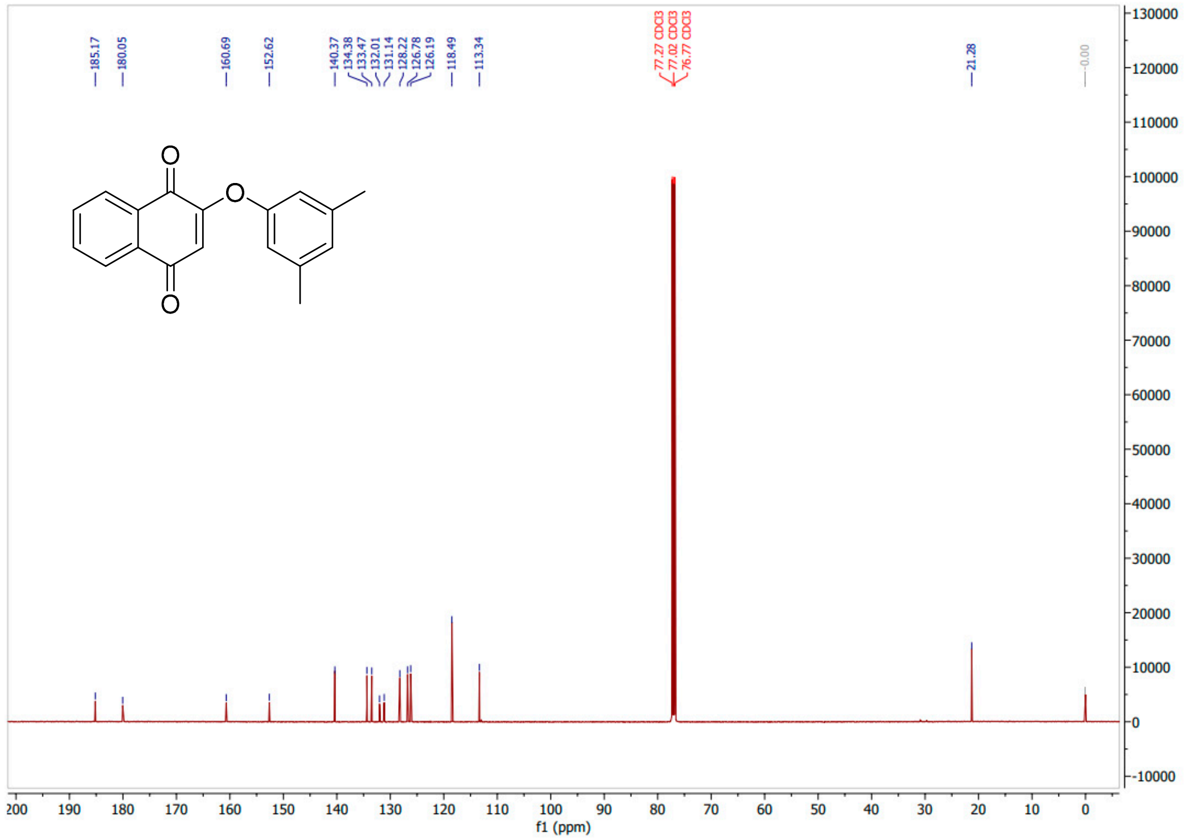

HRMS of 2-(3,5-dimethylphenoxy)naphthalene-1,4-dione (**5, 1-24**)

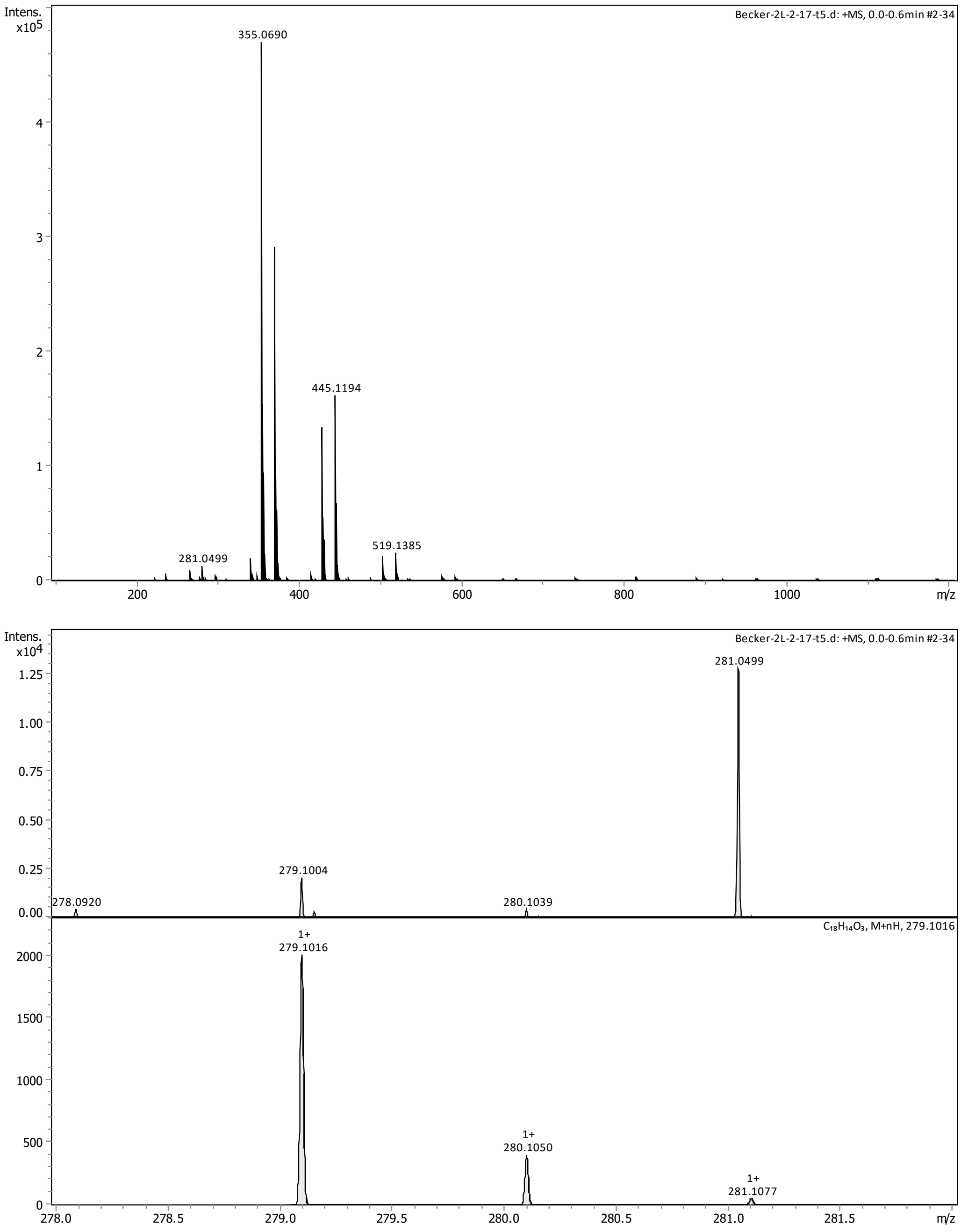

Meas. m/z	Ion formula	m/z	Chem. formula	Diff. (ppm)	Adduct ion
279.1004	$C_{18}H_{15}O_3$	279.1016	$C_{18}H_{14}O_3$	4.3	M+H

HPLC of 2-methyl-3-(4-(trifluoromethoxy)phenoxy)naphthalene-1,4-dione (**8, 1-20**)

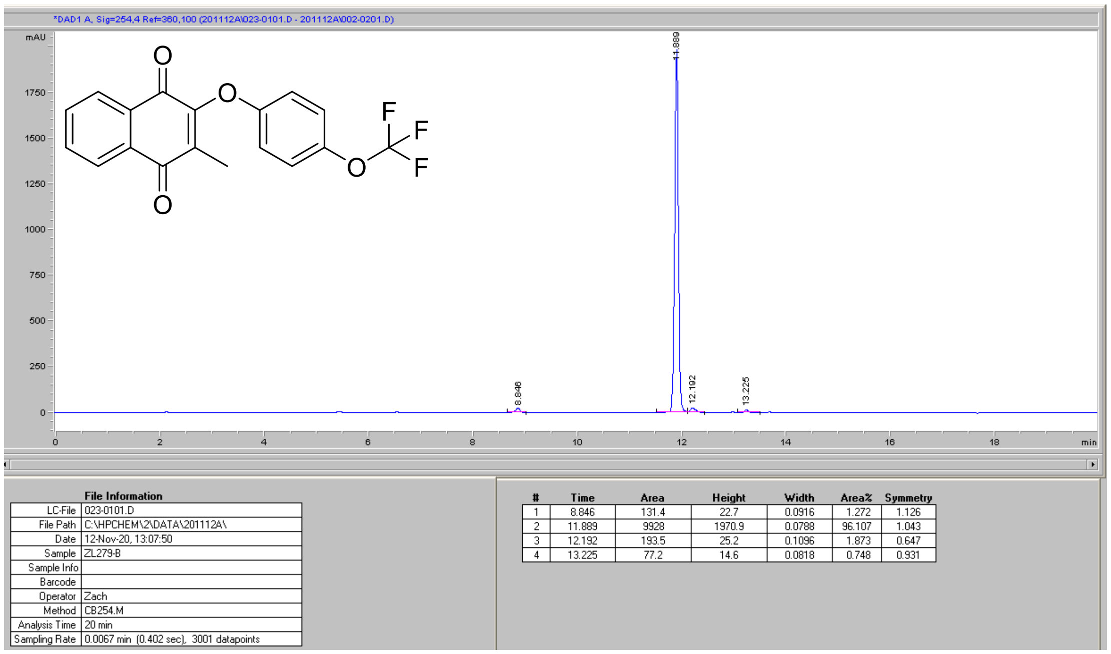

File Information

LC-File	023-0101.D
File Path	C:\HPCHEM\2\DATA\201112A\
Date	12-Nov-20, 13:07:50
Sample	ZL279-B
Sample Info	
Barcode	
Operator	Zach
Method	CB254.M
Analysis Time	20 min
Sampling Rate	0.0067 min (0.402 sec), 3001 datapoints

#	Time	Area	Height	Width	Area%	Symmetry
1	8.846	131.4	22.7	0.0916	1.272	1.126
2	11.889	9928	1970.9	0.0788	96.107	1.043
3	12.192	193.5	25.2	0.1096	1.873	0.647
4	13.225	77.2	14.6	0.0818	0.748	0.931

^{1}H NMR of 2-methyl-3-(4-(trifluoromethoxy)phenoxy)naphthalene-1,4-dione (**8, 1-20**)

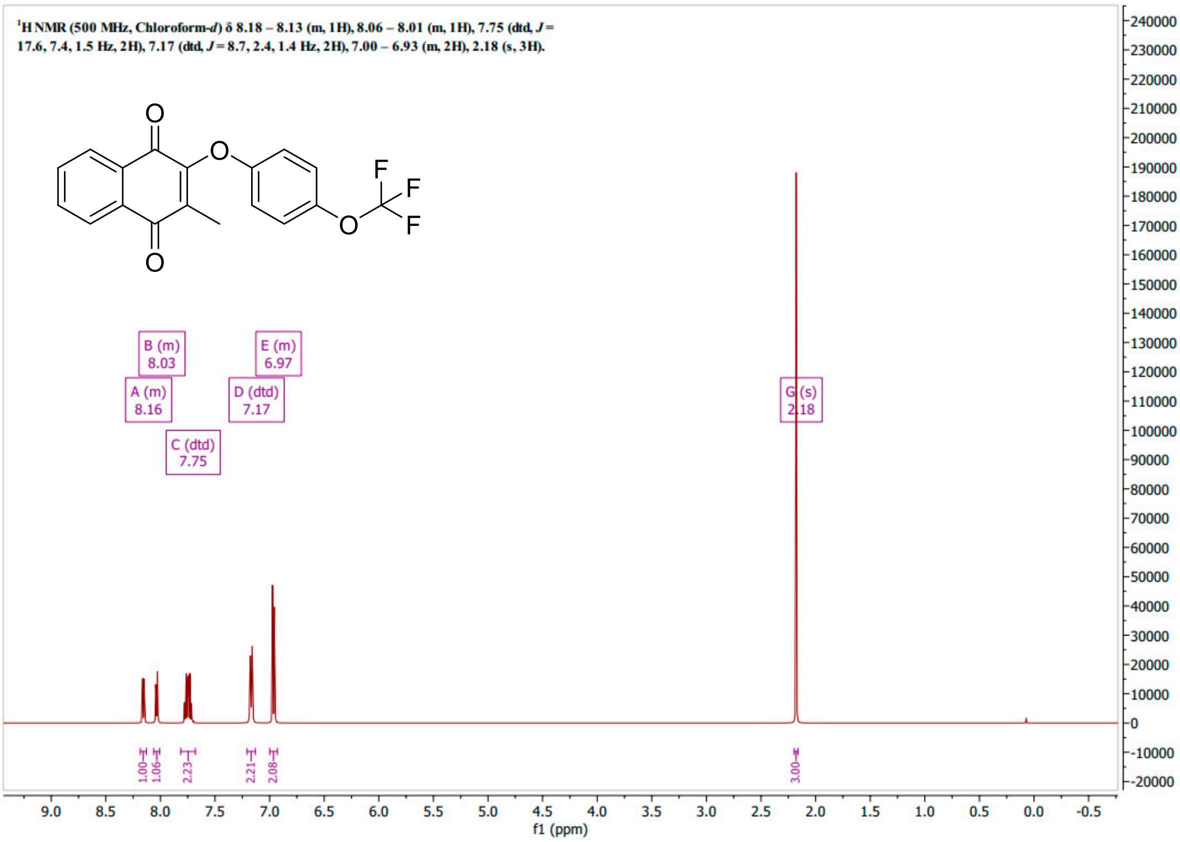

^{13}C NMR of 2-methyl-3-(4-(trifluoromethoxy)phenoxy)naphthalene-1,4-dione (**8, 1-20**)

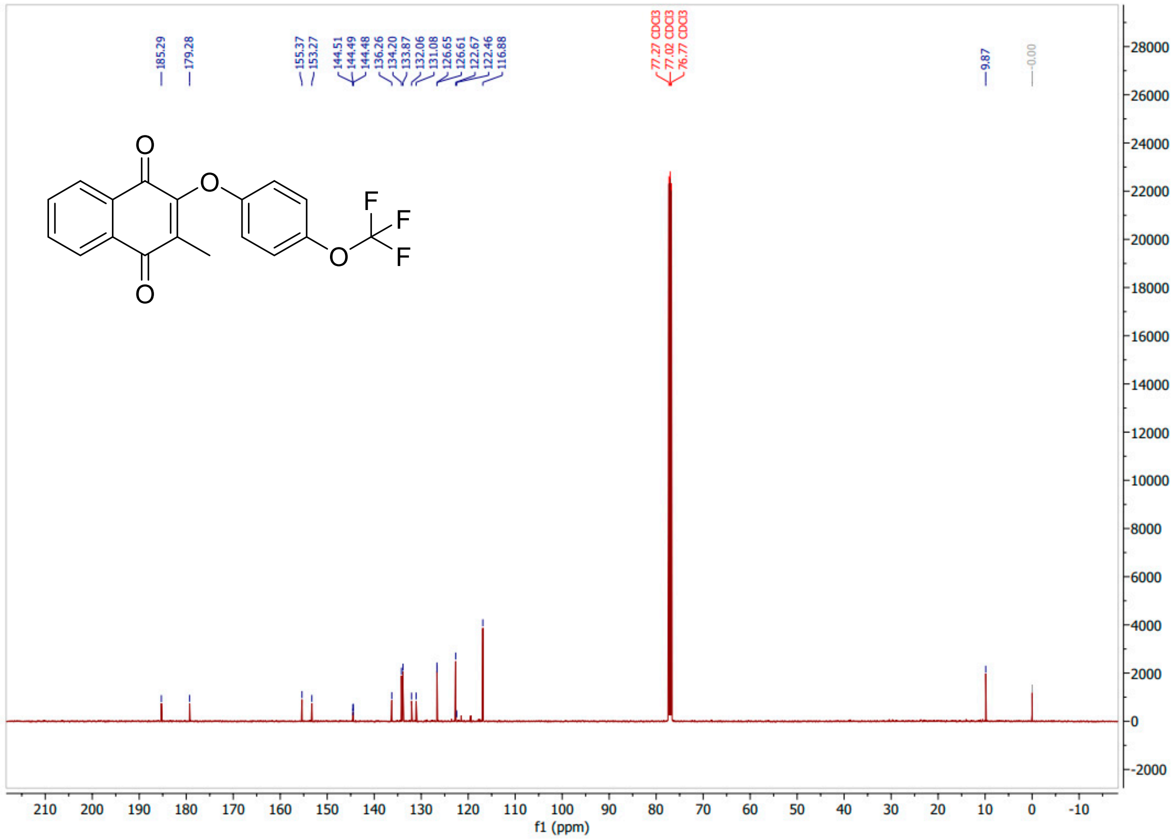

HRMS of 2-methyl-3-(4-(trifluoromethoxy)phenoxy)naphthalene-1,4-dione (**8, 1-20**)

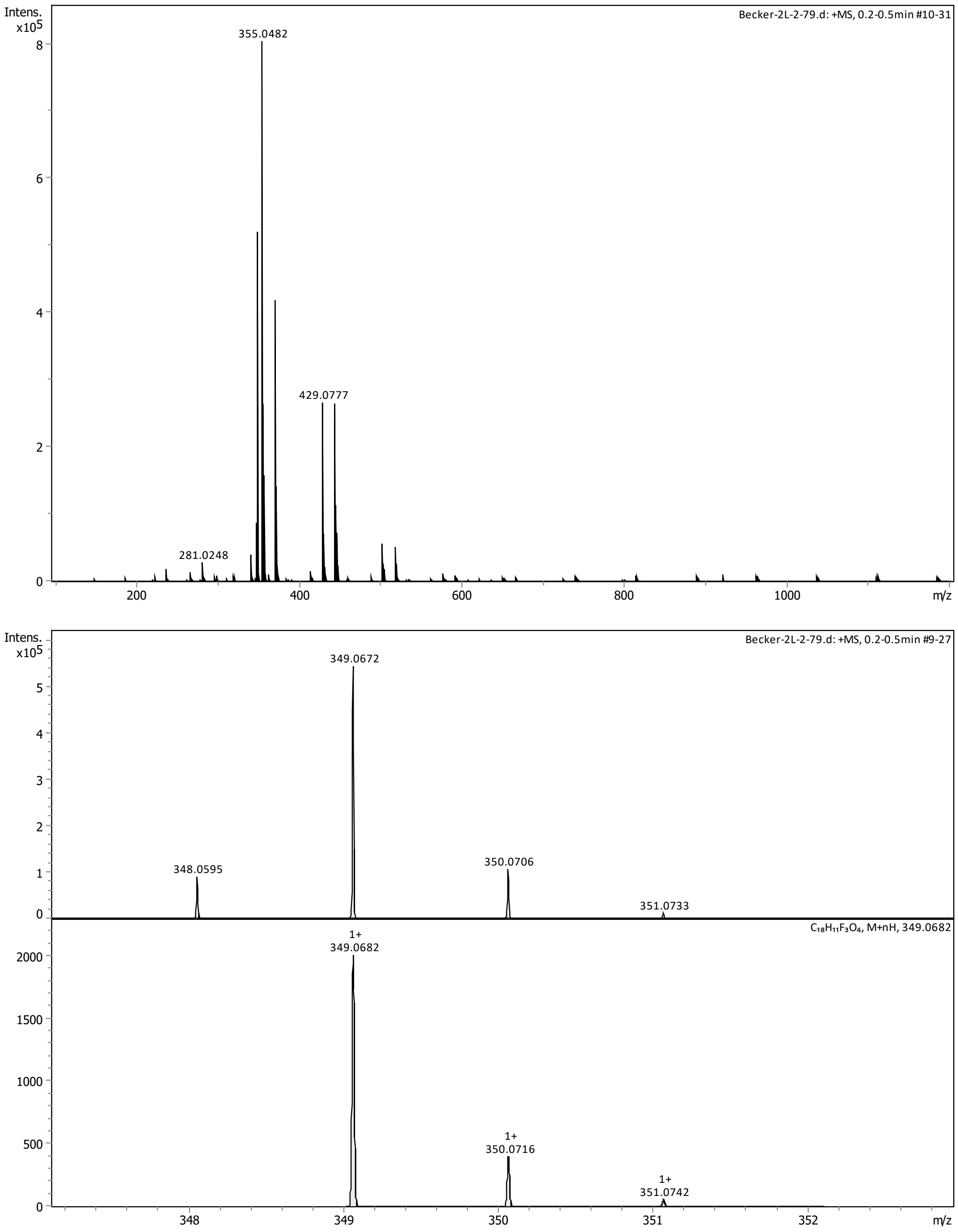

Meas. m/z	Ion formula	m/z	Chem. formula	Diff. (ppm)	Adduct ion
349.0672	$C_{18}H_{12}F_3O_4$	349.0682	$C_{18}H_{11}F_3O_4$	2.86	M+H

HPLC of 2-(4-fluorophenoxy)-3-methylnaphthalene-1,4-dione (**9, 1-22**)

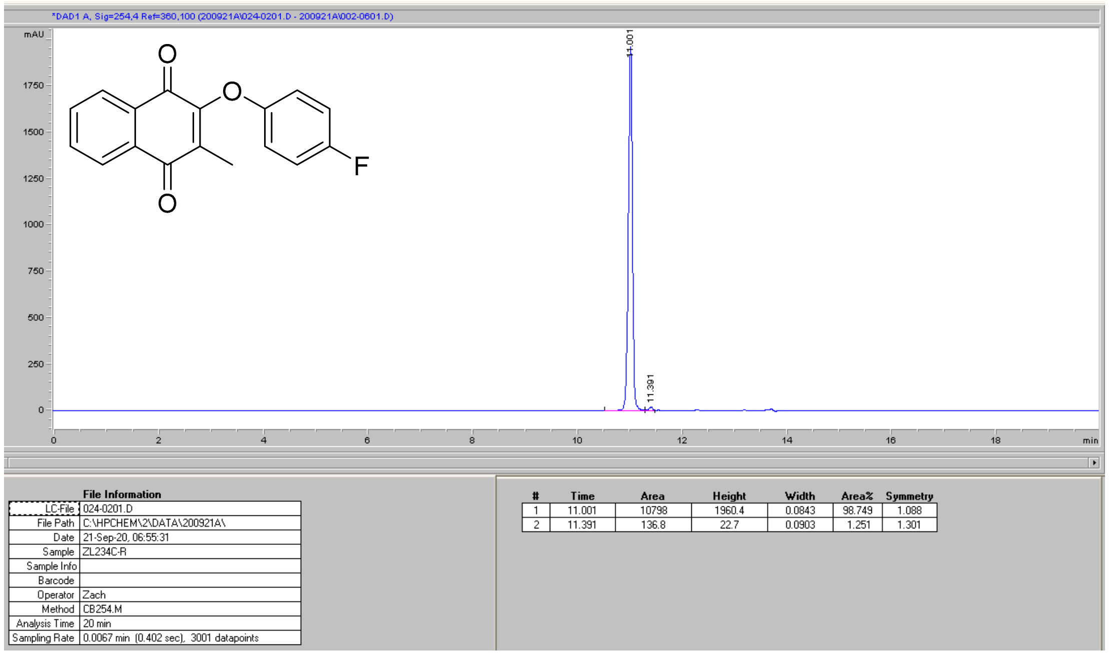

File Information

LC-File	024-0201.D
File Path	C:\HPCHEM\2\DATA\200921A\
Date	21-Sep-20, 06:55:31
Sample	ZL234C-R
Sample Info	
Barcode	
Operator	Zach
Method	CB254.M
Analysis Time	20 min
Sampling Rate	0.0067 min (0.402 sec), 3001 datapoints

#	Time	Area	Height	Width	Area%	Symmetry
1	11.001	10798	1960.4	0.0843	98.749	1.088
2	11.391	136.8	22.7	0.0903	1.251	1.301

^{1}H NMR of 2-(4-fluorophenoxy)-3-methylnaphthalene-1,4-dione (**9, 1-22**)

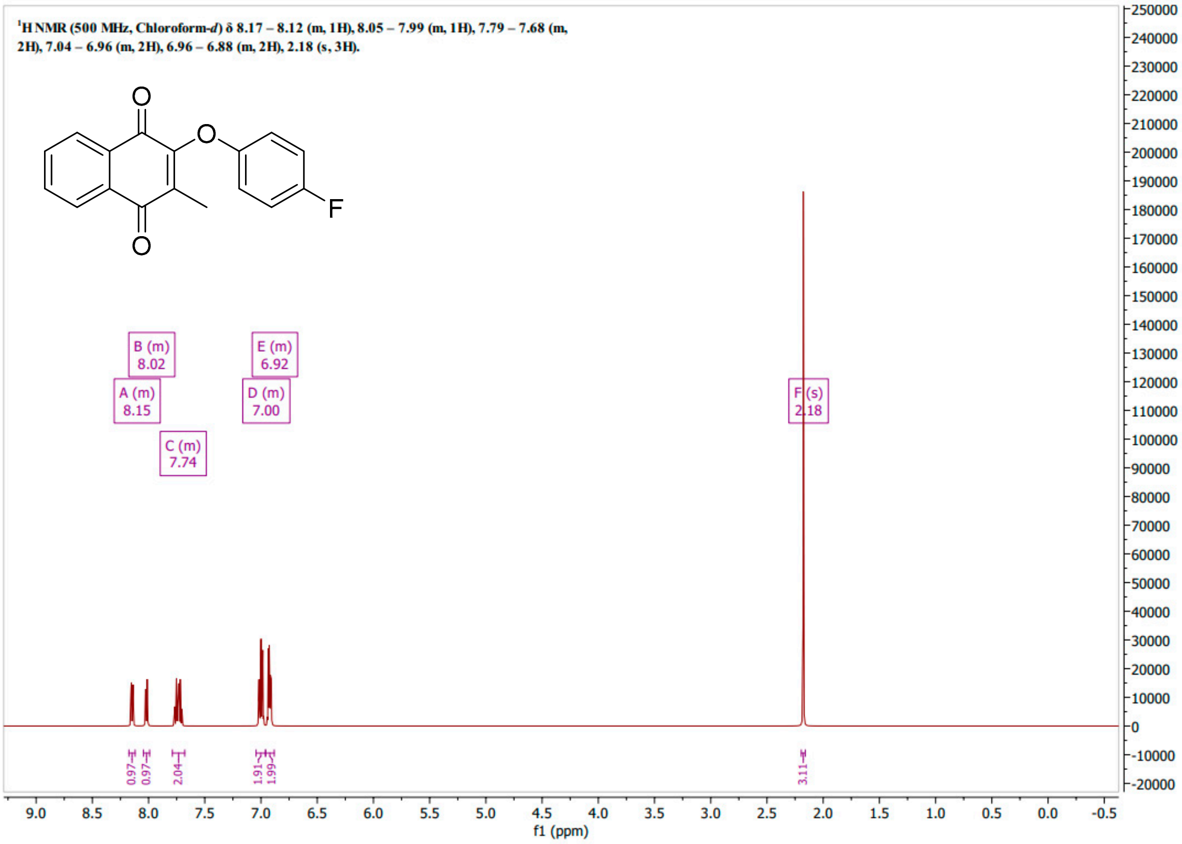

^{13}C NMR of 2-(4-fluorophenoxy)-3-methylnaphthalene-1,4-dione (**9, 1-22**)

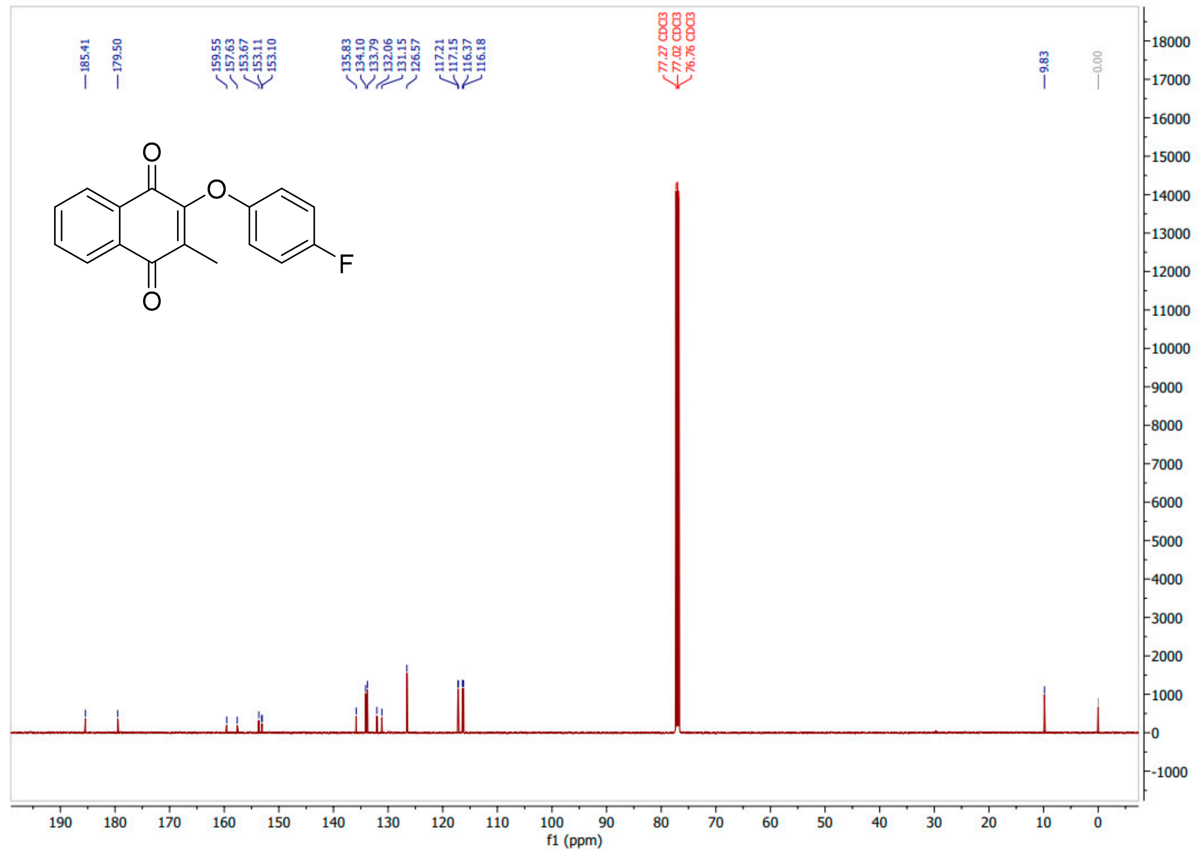

HRMS of 2-(4-fluorophenoxy)-3-methylnaphthalene-1,4-dione (**9, 1-22**)

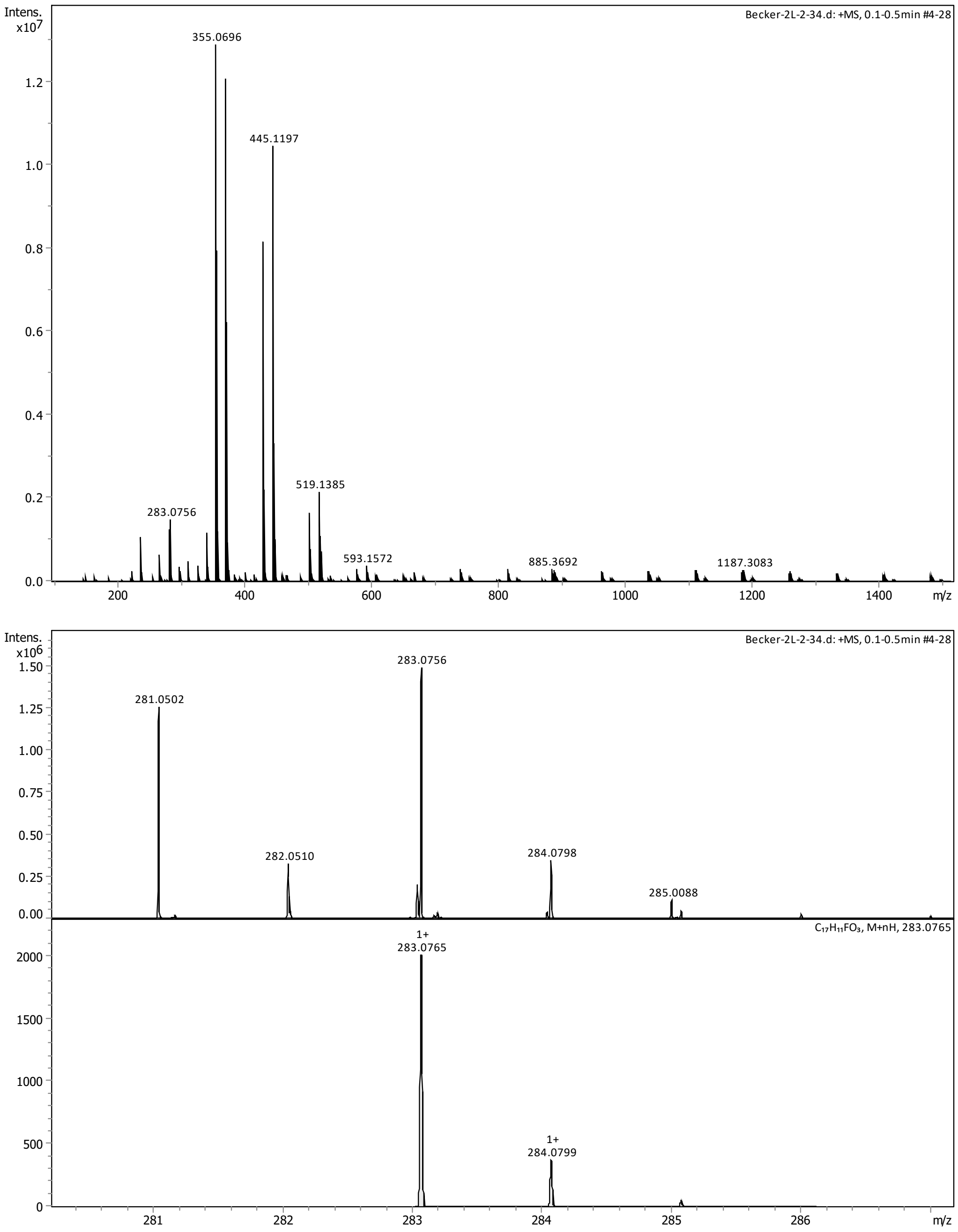

Meas. m/z	Ion formula	m/z	Chem. formula	Diff. (ppm)	Adduct ion
283.0756	$C_{17}H_{12}FO_3$	283.0765	$C_{17}H_{11}FO_3$	3.53	M+H

HPLC of 2-((2-(4-(trifluoromethoxy)phenoxy)phenyl)amino)naphthalene-1,4-dione (**12, 1-21**)

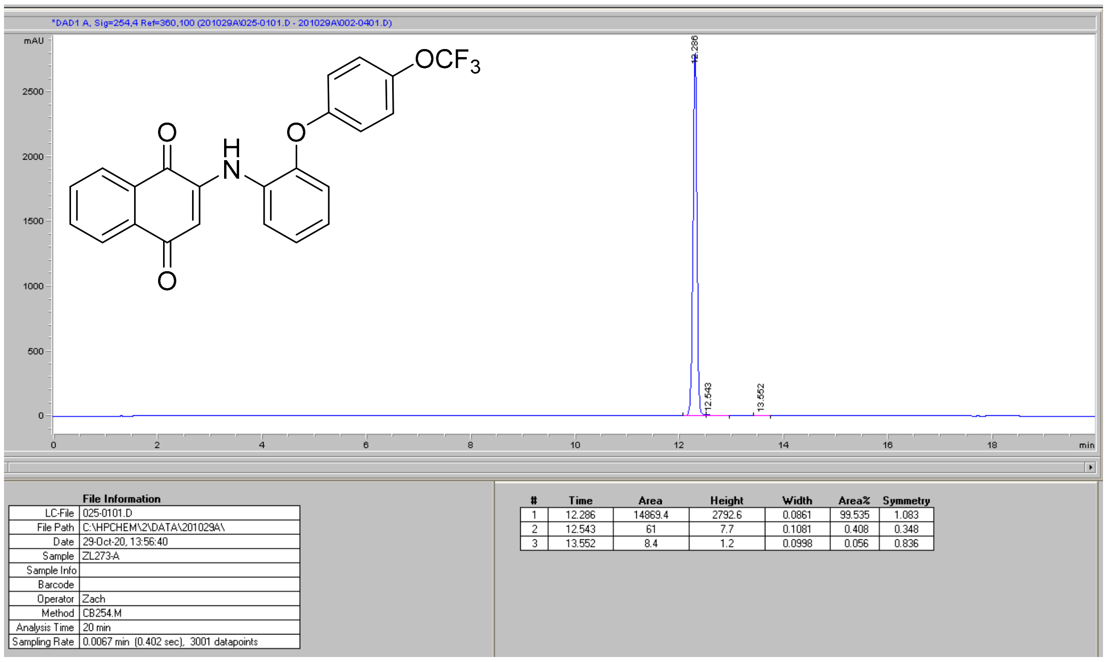

File Information

LC-File	025-0101.D
File Path	C:\HPCHEM\2\DATA\201029A\
Date	29-Oct-20, 13:56:40
Sample	ZL273-A
Sample Info	
Barcode	
Operator	Zach
Method	CB254.M
Analysis Time	20 min
Sampling Rate	0.0067 min (0.402 sec), 3001 datapoints

#	Time	Area	Height	Width	Area%	Symmetry
1	12.286	14869.4	2792.6	0.0861	99.535	1.083
2	12.543	61	7.7	0.1081	0.408	0.348
3	13.552	8.4	1.2	0.0998	0.056	0.836

^{1}H NMR of 2-((2-(4-(trifluoromethoxy)phenoxy)phenyl)amino)naphthalene-1,4-dione (**12, 1-21**)

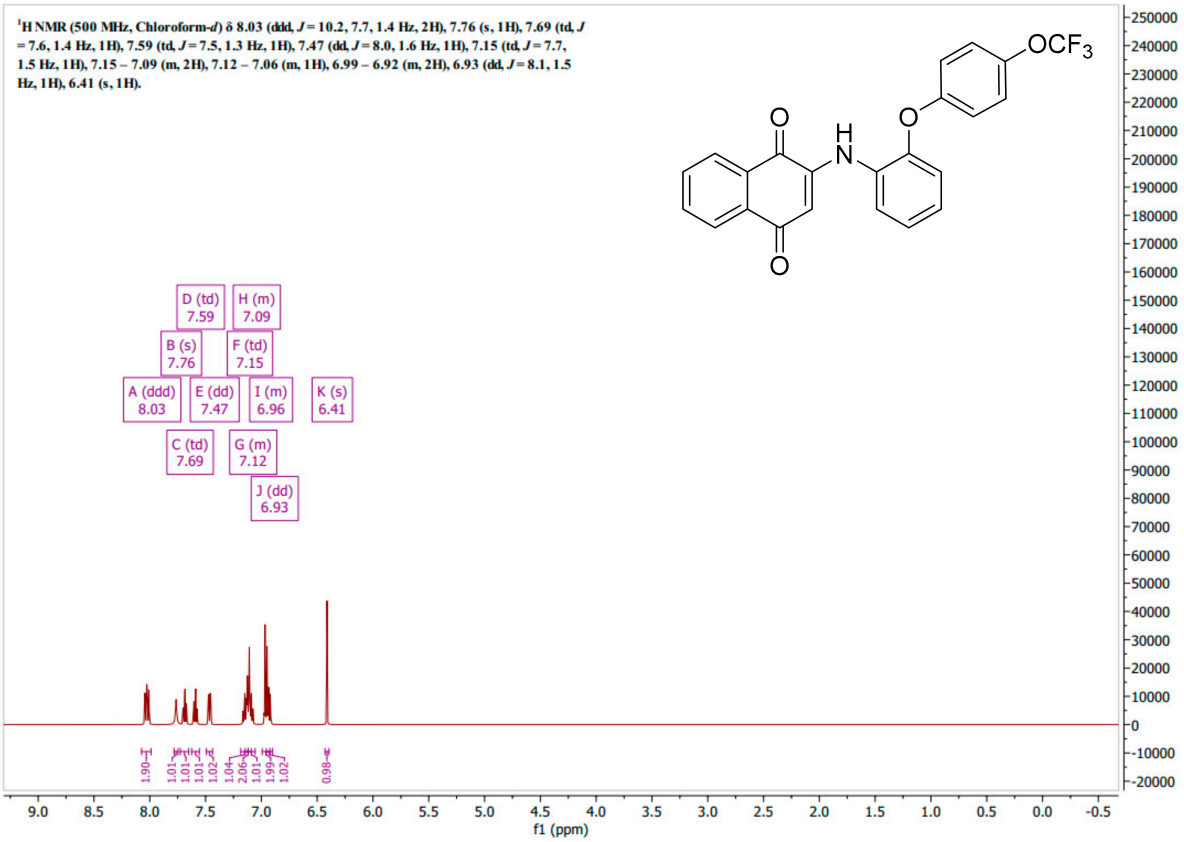

^{13}C NMR of 2-((2-(4-(trifluoromethoxy)phenoxy)phenyl)amino)naphthalene-1,4-dione (**12, 1-21**)

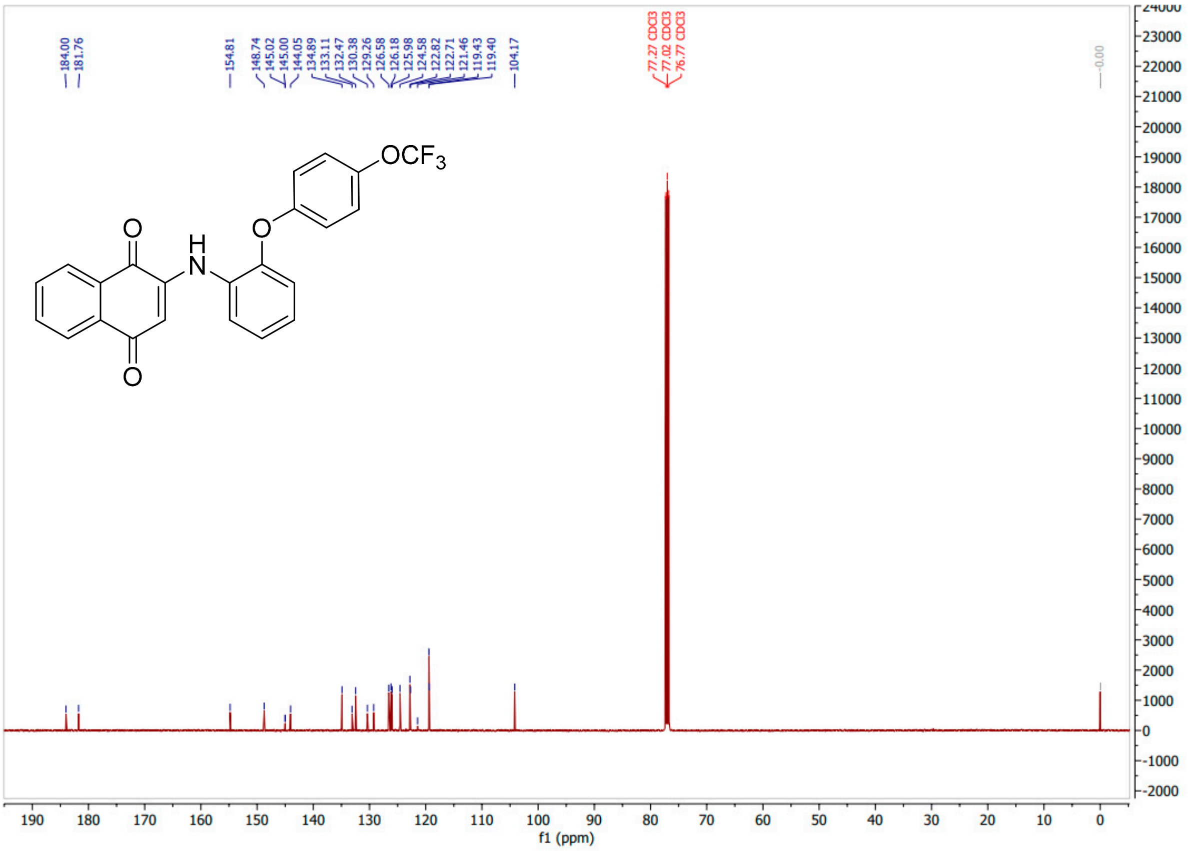

HRMS of 2-((2-(4-(trifluoromethoxy)phenoxy)phenyl)amino)naphthalene-1,4-dione (**12, 1-21**)

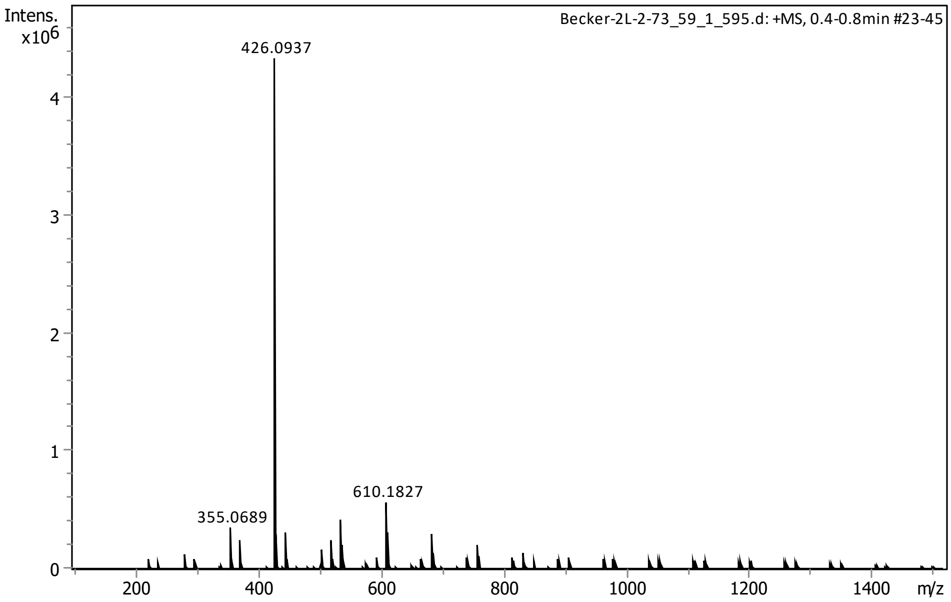

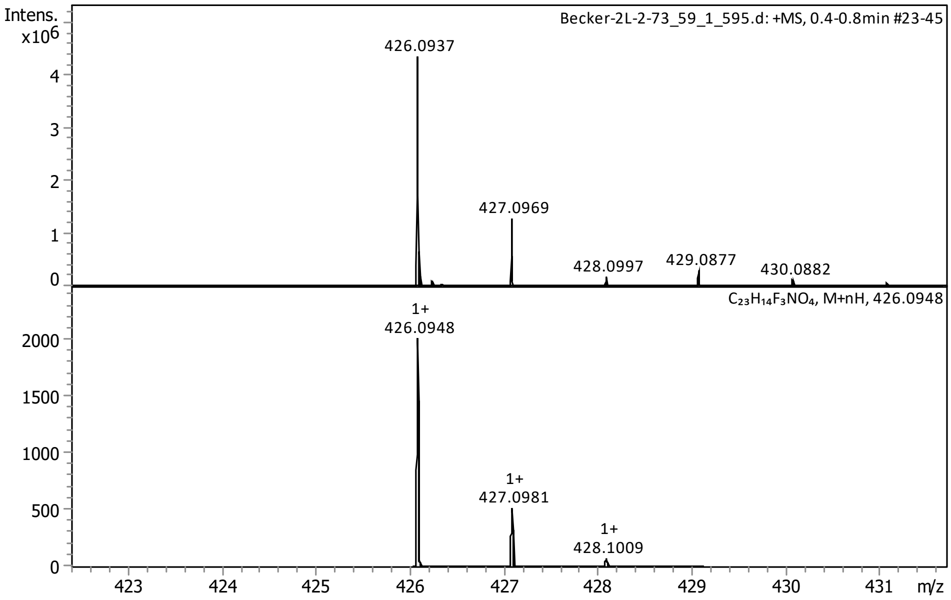

Meas. m/z	Ion formula	m/z	Chem. formula	Diff. (ppm)	Adduct ion
426.0937	$C_{23}H_{15}F_3NO_4$	426.0948	$C_{23}H_{14}F_3NO_4$	2.58	M+H

HPLC of N-(4-(2-((1,4-dioxo-1,4-dihydronaphthalen-2-yl)amino)phenoxy)phenyl)acetamide (**13, 1-16**)

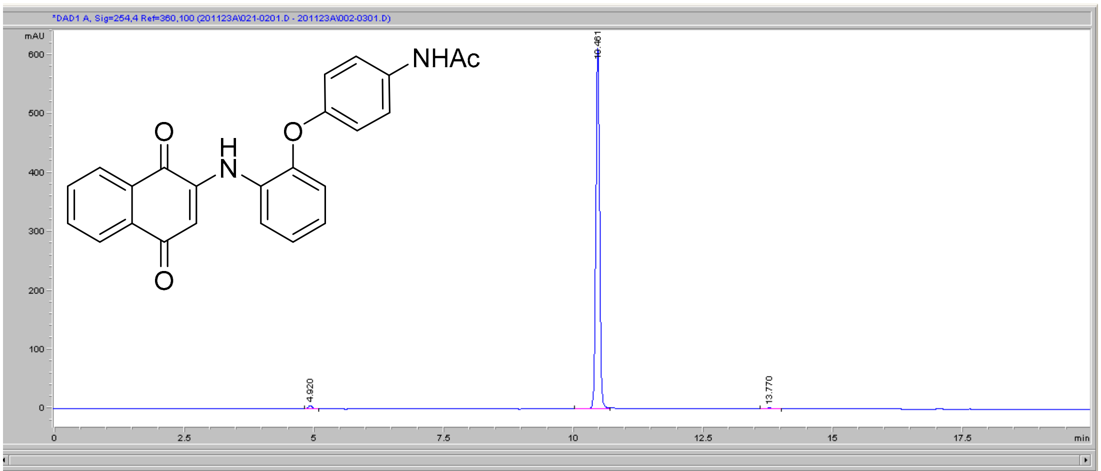

File Information

LC-File	021-0201.D
File Path	C:\HPCHEM\2\DATA\201123A\
Date	23-Nov-20, 07:39:14
Sample	ZL283-R
Sample Info	
Barcode	
Operator	Zach
Method	CB254.M
Analysis Time	20 min
Sampling Rate	0.0067 min (0.402 sec), 3001 datapoints

#	Time	Area	Height	Width	Area%	Symmetry
1	4.92	30.8	5.2	0.0893	0.981	0.847
2	10.461	3083.9	607.4	0.0792	98.382	0.987
3	13.77	20	1.9	0.1457	0.637	0.924

^{1}H NMR of N-(4-(2-((1,4-dioxo-1,4-dihydronaphthalen-2-yl)amino)phenoxy)phenyl)acetamide (**13, 1-16**)

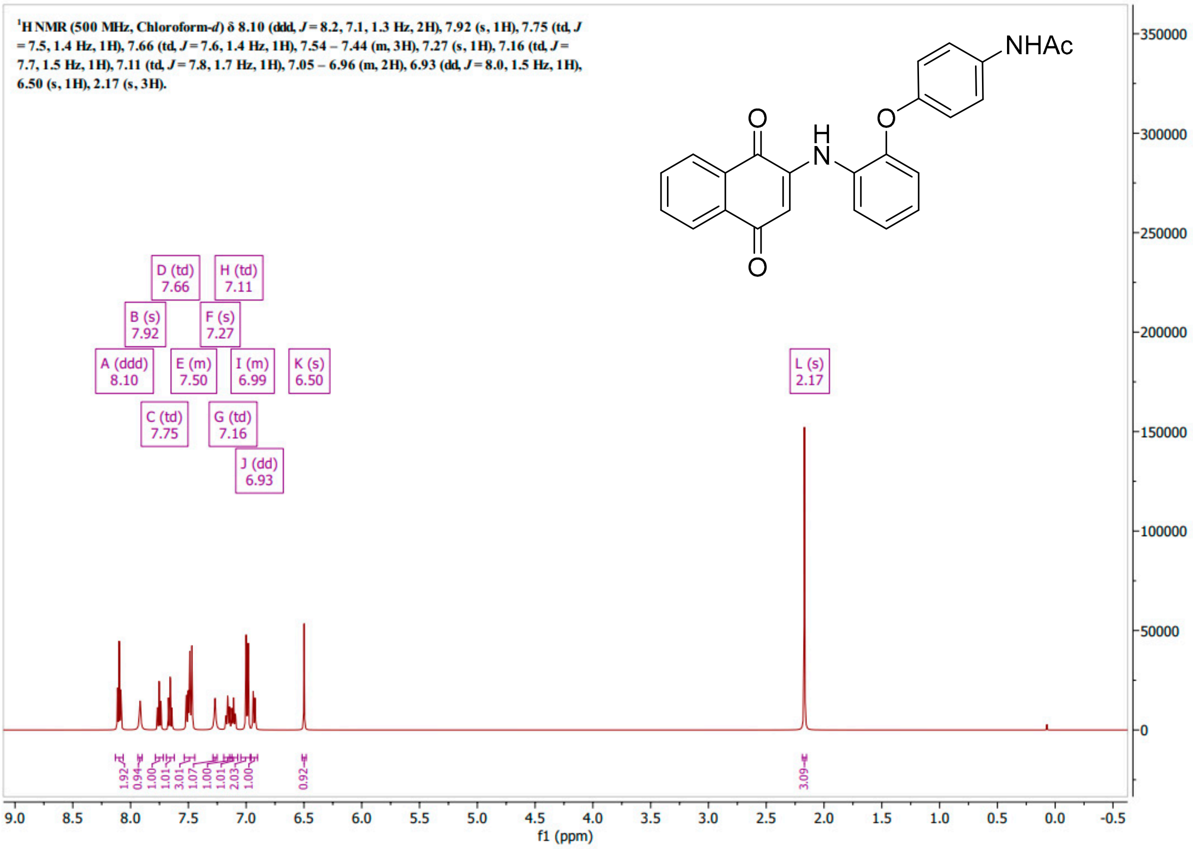

^{13}C NMR of N-(4-(2-((1,4-dioxo-1,4-dihydronaphthalen-2-yl)amino)phenoxy)phenyl)acetamide (**13, 1-16**)

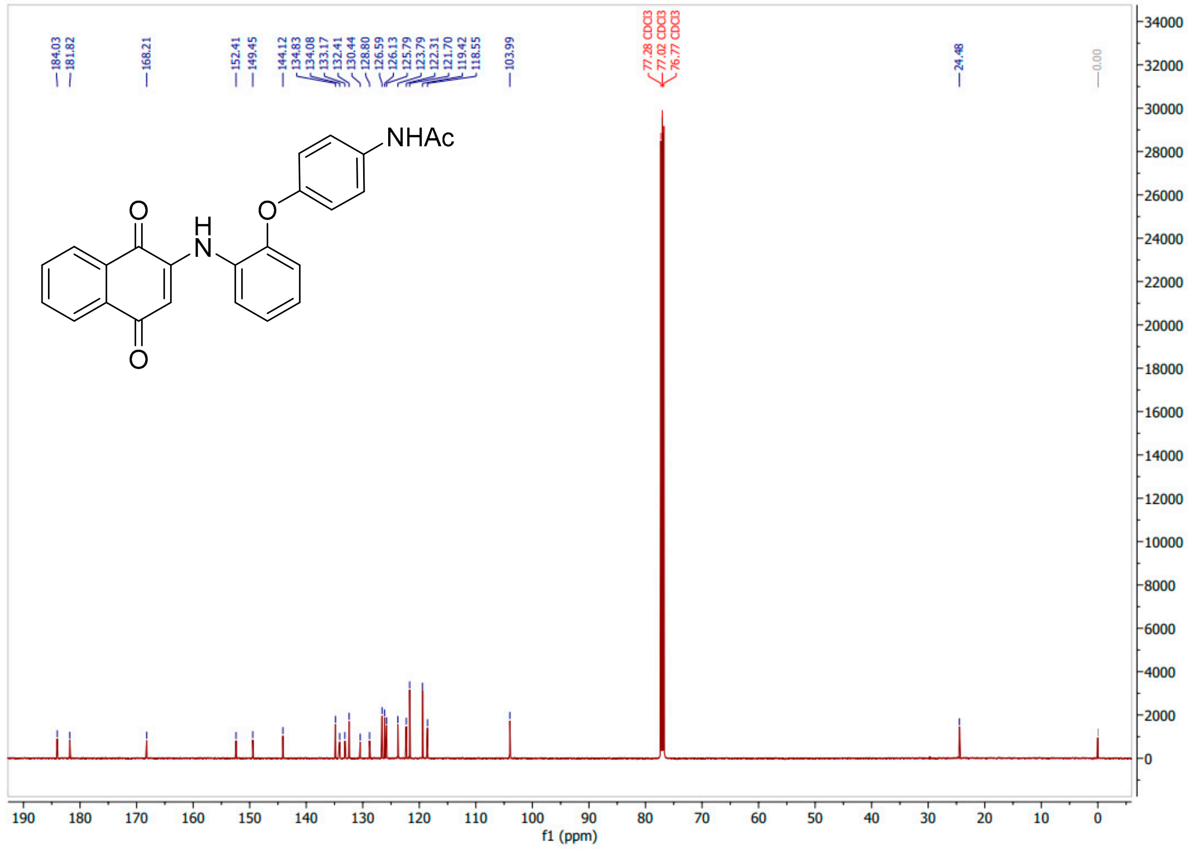

HRMS of N-(4-(2-((1,4-dioxo-1,4-dihydronaphthalen-2-yl)amino)phenoxy)phenyl)acetamide (**13, 1-16**)

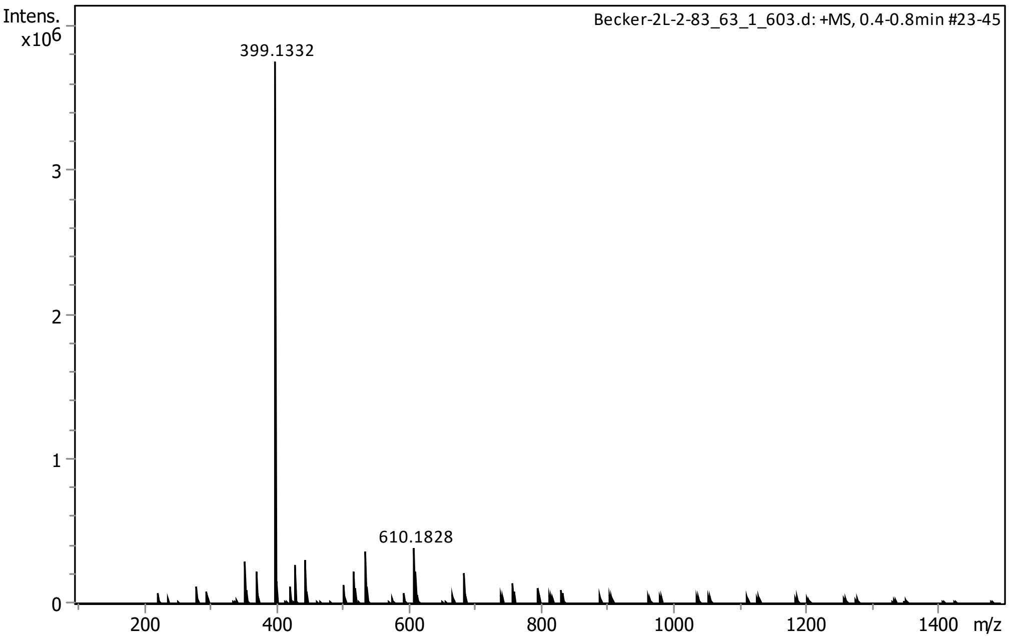

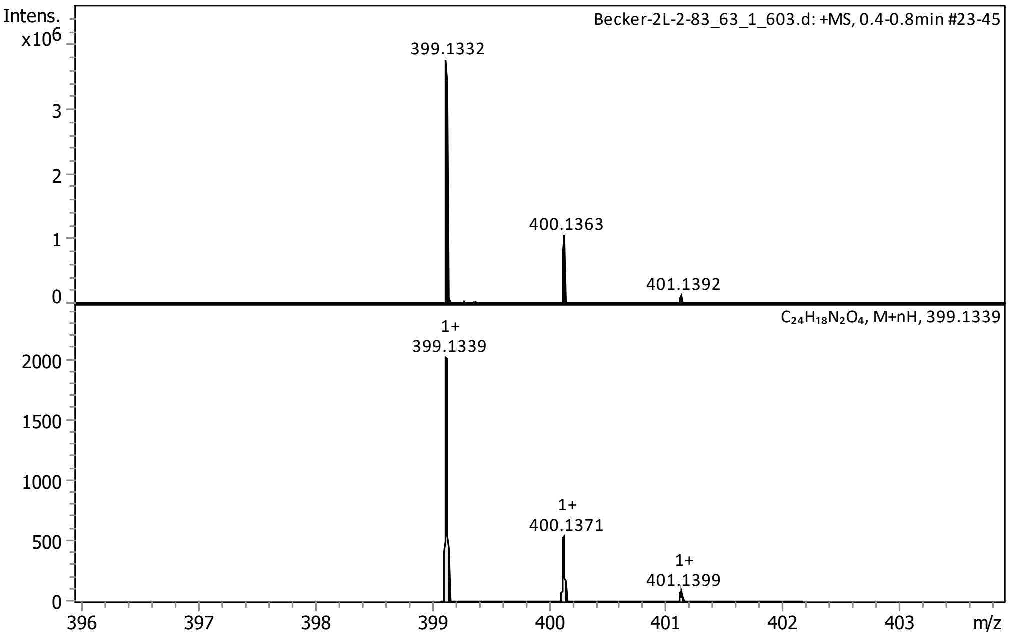

Meas. m/z	Ion formula	m/z	Chem. formula	Diff. (ppm)	Adduct ion
399.1332	$C_{24}H_{19}N_2O_4$	399.1339	$C_{24}H_{18}N_2O_4$	1.75	M+H

HPLC of 2-methyl-3-((2-(4-(trifluoromethoxy)phenoxy)phenyl)amino)naphthalene-1,4-dione (**14, 1-19**)

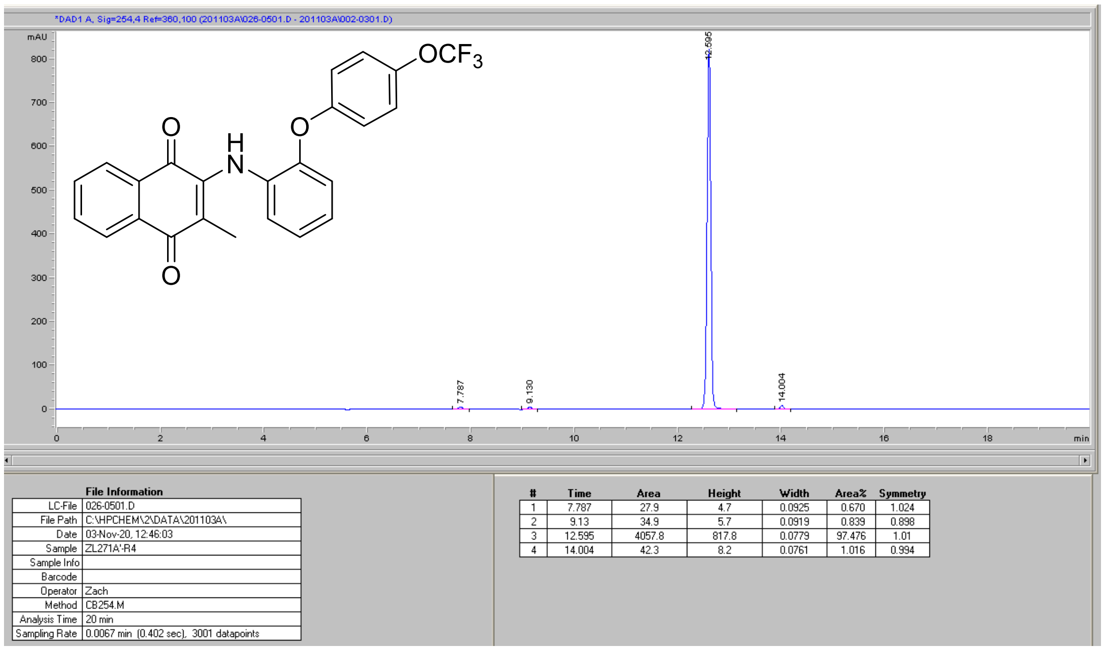

File Information	
LC-File	026-0501.D
File Path	C:\HPCHEM\2\DATA\201103A\
Date	03-Nov-20, 12:46:03
Sample	ZL271A'-R4
Sample Info	
Barcode	
Operator	Zach
Method	CB254.M
Analysis Time	20 min
Sampling Rate	0.0067 min (0.402 sec), 3001 datapoints

#	Time	Area	Height	Width	Area%	Symmetry
1	7.787	27.9	4.7	0.0925	0.670	1.024
2	9.13	34.9	5.7	0.0919	0.839	0.898
3	12.595	4057.8	817.8	0.0779	97.476	1.01
4	14.004	42.3	8.2	0.0761	1.016	0.994

^{1}H NMR of 2-methyl-3-((2-(4-(trifluoromethoxy)phenoxy)phenyl)amino)naphthalene-1,4-dione (**14, 1-19**)

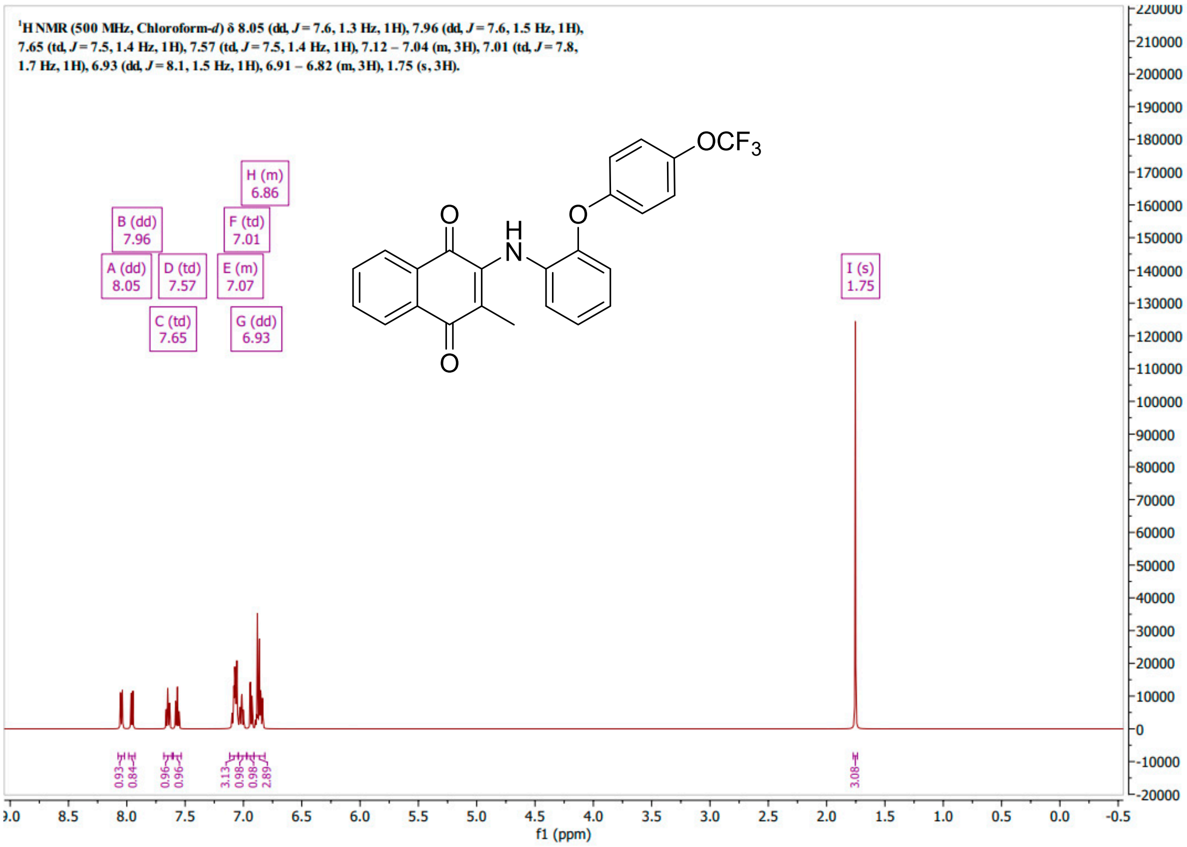

^{13}C NMR of 2-methyl-3-((2-(4-(trifluoromethoxy)phenoxy)phenyl)amino)naphthalene-1,4-dione (**14, 1-19**)

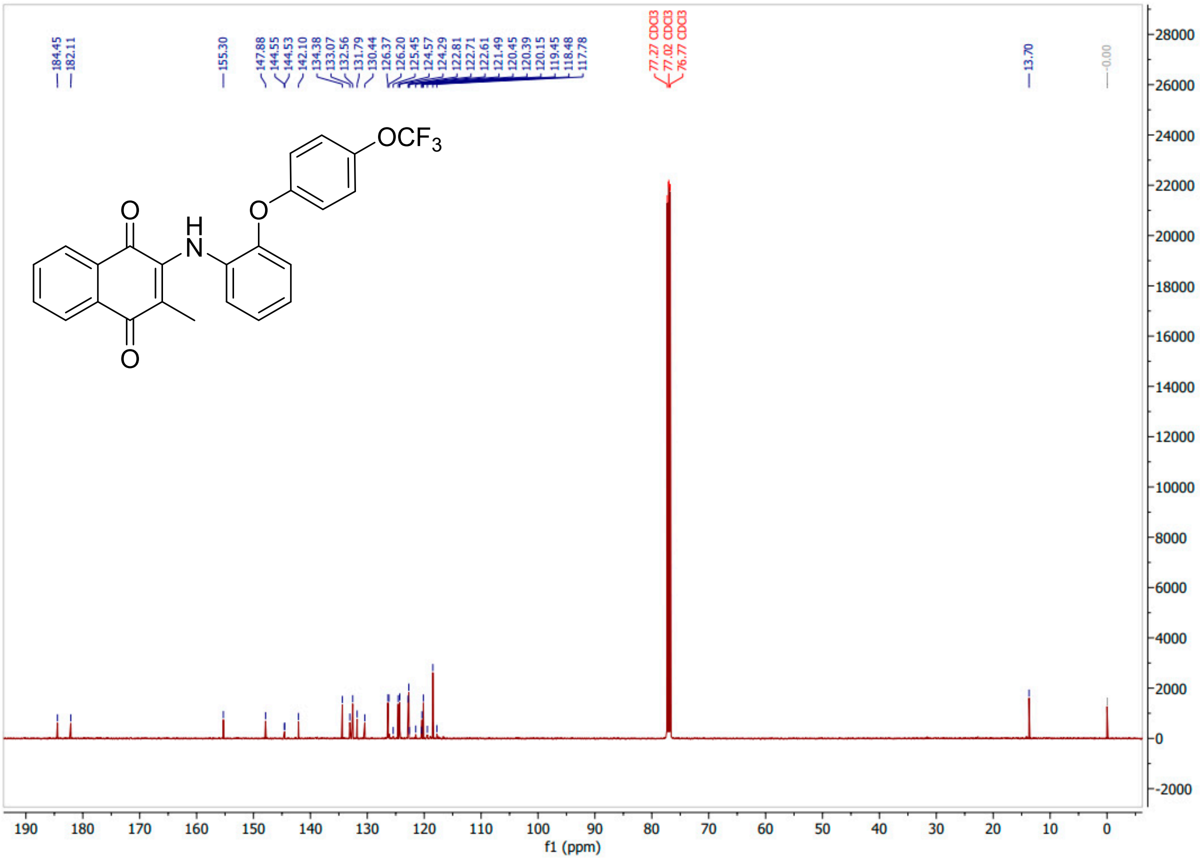

HRMS of 2-methyl-3-((2-(4-(trifluoromethoxy)phenoxy)phenyl)amino)naphthalene-1,4-dione (**14, 1-19**)

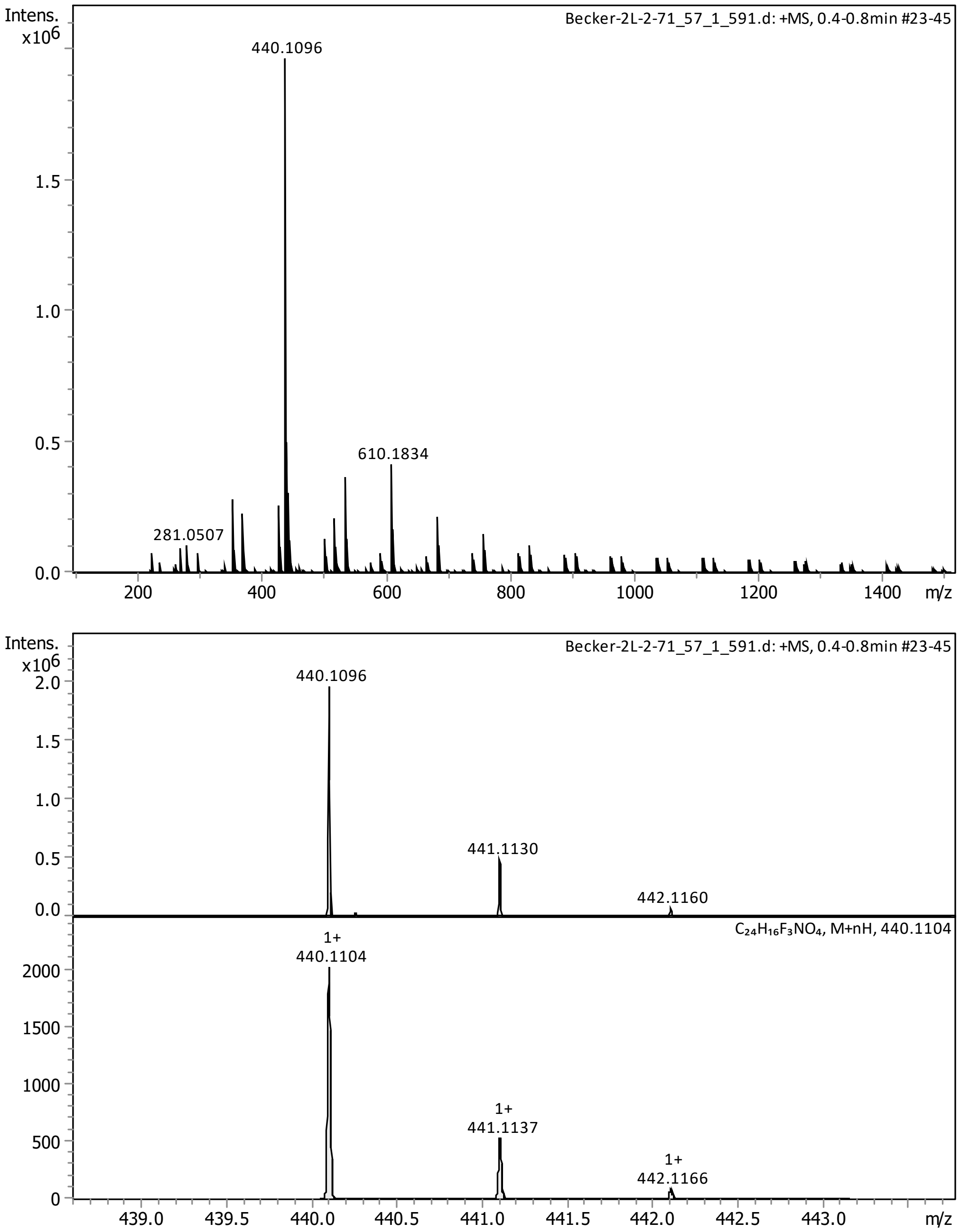

Meas. m/z	Ion formula	m/z	Chem. formula	Diff. (ppm)	Adduct ion
440.1096	$C_{24}H_{17}F_3NO_4$	440.1104	$C_{24}H_{16}F_3NO_4$	1.82	M+H

HPLC of N-(4-(2-((3-methyl-1,4-dioxo-1,4-dihydronaphthalen-2-yl)amino)phenoxy)phenyl)acetamide (**15, 1-31**)

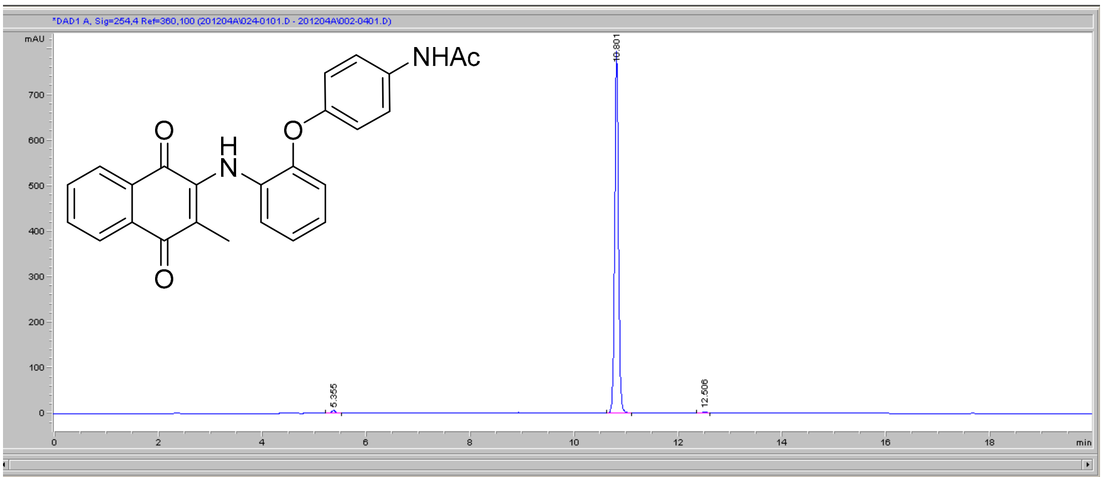

File Information

LC-File	024-0101.D
File Path	C:\HPCHEM\2\DATA\201204A\
Date	04-Dec-20, 08:35:52
Sample	
Sample Info	
Barcode	
Operator	Zach
Method	CB254.M
Analysis Time	20 min
Sampling Rate	0.0067 min (0.402 sec), 3001 datapoints

#	Time	Area	Height	Width	Area%	Symmetry
1	5.355	37.1	6.3	0.0924	0.902	0.961
2	10.801	4053.7	792.1	0.0797	98.583	0.951
3	12.506	21.2	3.3	0.0946	0.515	1.232

^{1}H NMR of N-(4-(2-((3-methyl-1,4-dioxo-1,4-dihydronaphthalen-2-yl)amino)phenoxy)phenyl)acetamide (**15, 1-31**)

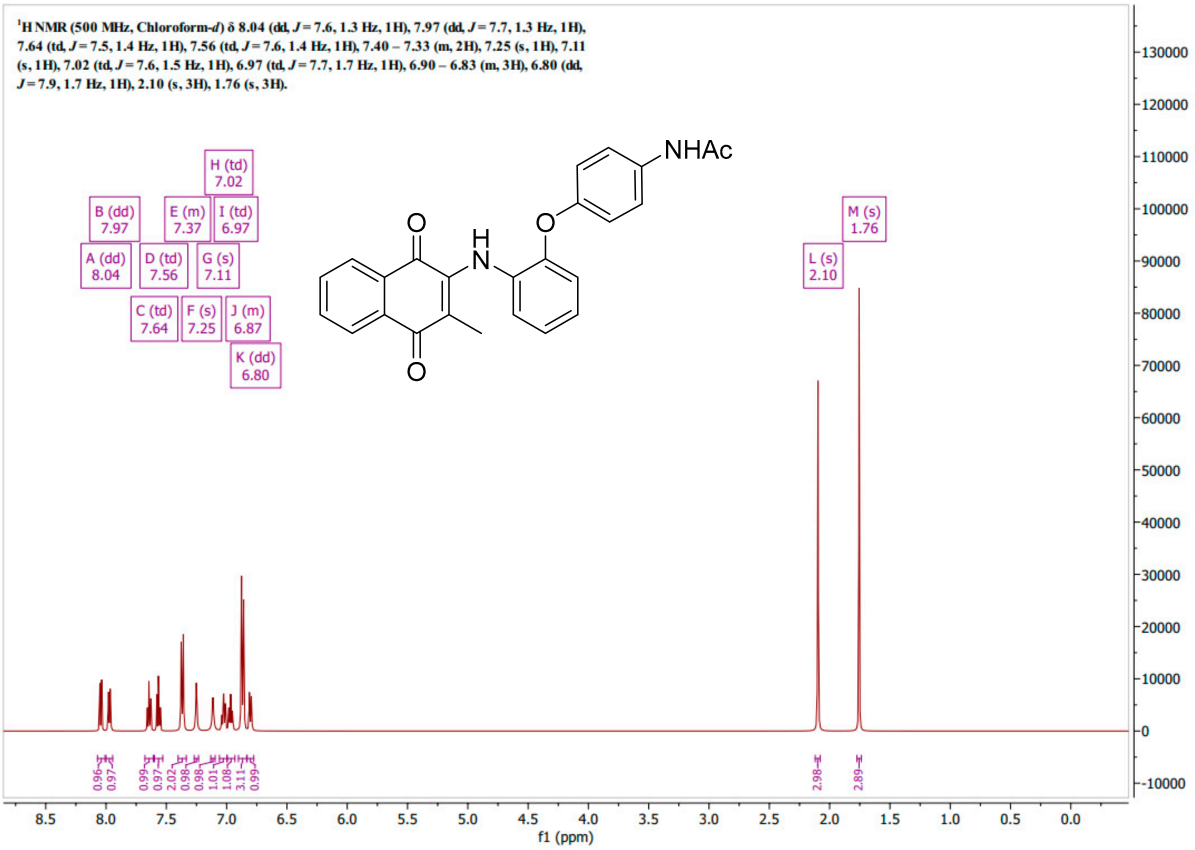

^{13}C NMR of N-(4-(2-((3-methyl-1,4-dioxo-1,4-dihydronaphthalen-2-yl)amino)phenoxy)phenyl)acetamide (**15, 1-31**)

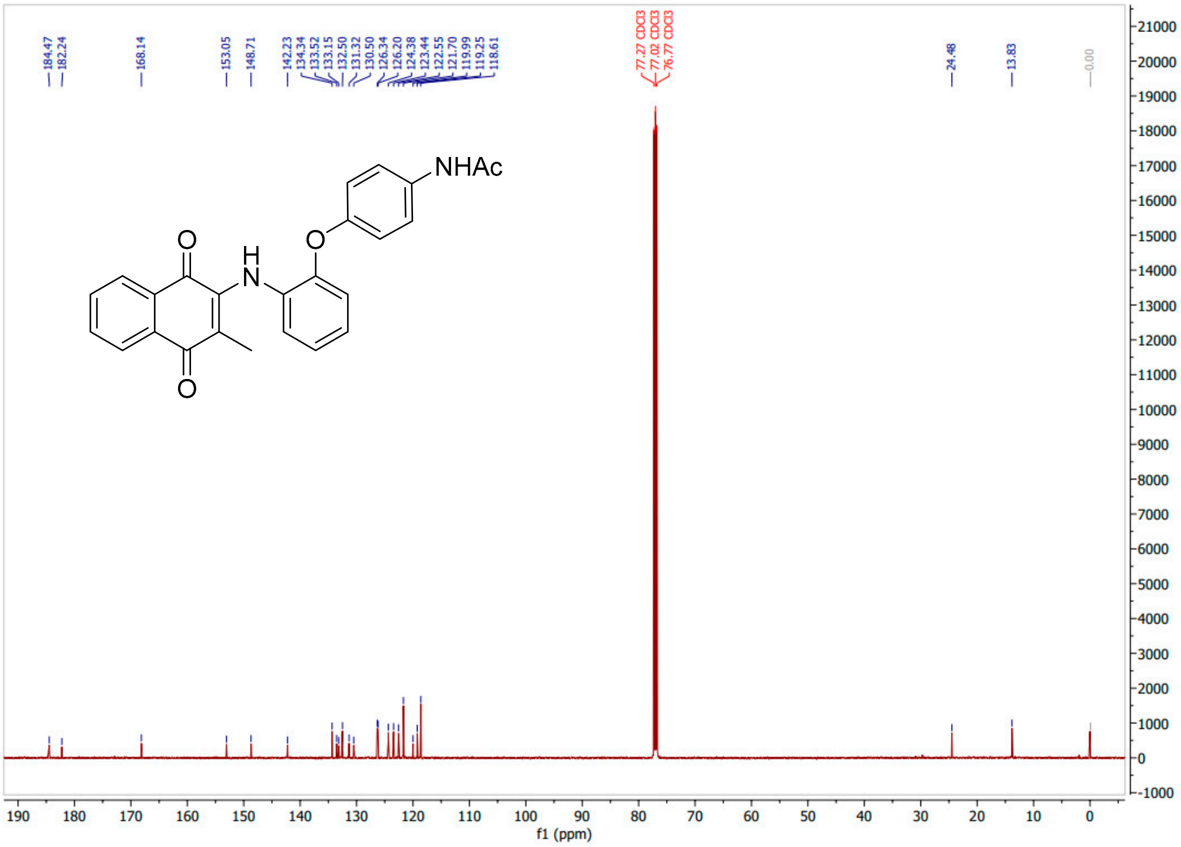

HRMS of N-(4-(2-((3-methyl-1,4-dioxo-1,4-dihydronaphthalen-2-yl)amino)phenoxy)phenyl)acetamide (**15, 1-31**)

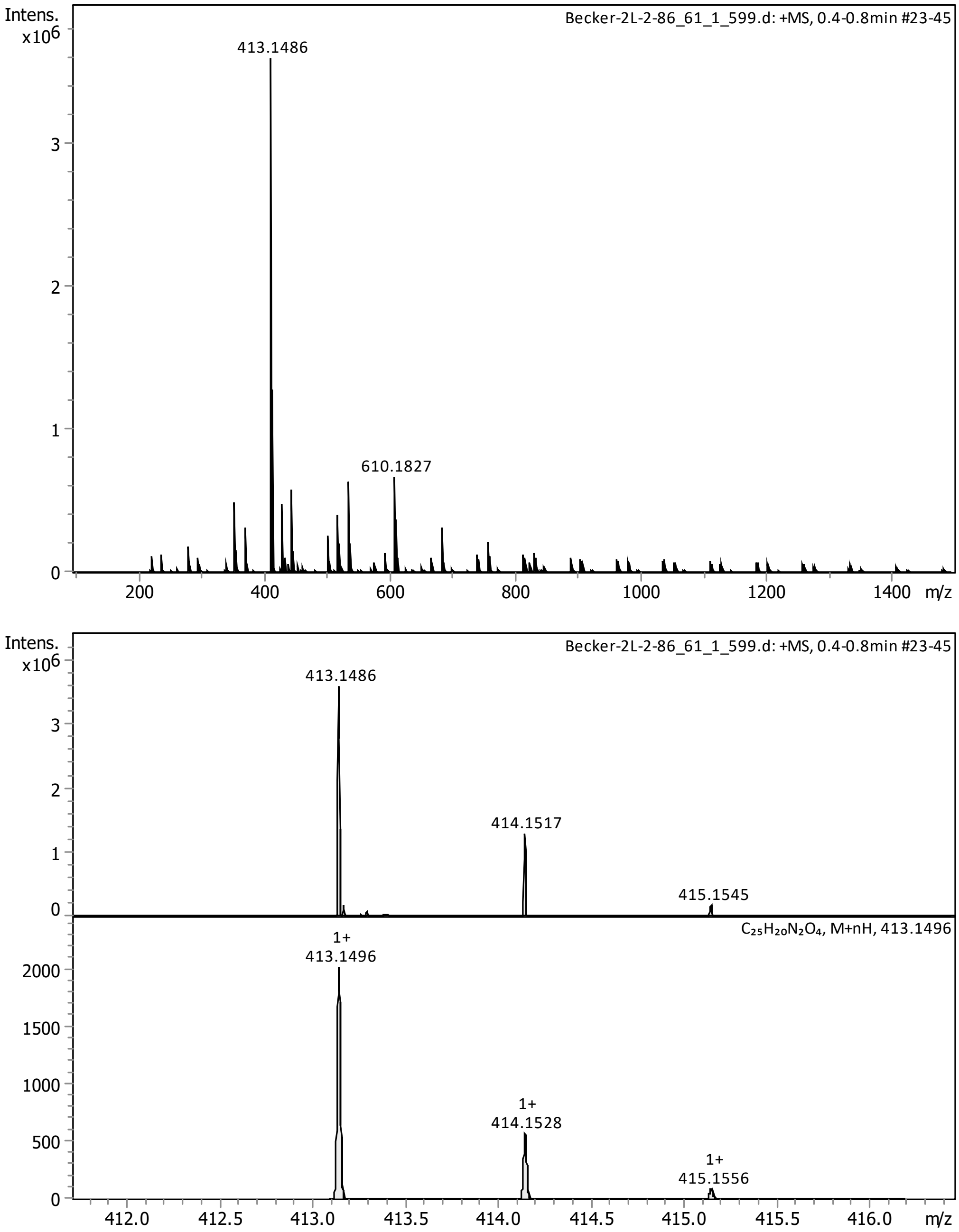

Meas. m/z	Ion formula	m/z	Chem. formula	Diff. (ppm)	Adduct ion
413.1486	$C_{25}H_{21}N_2O_4$	413.1496	$C_{25}H_{20}N_2O_4$	2.41	M+H

HPLC of 2-((4-(4-(trifluoromethoxy)phenoxy)phenyl)amino)naphthalene-1,4-dione (**16, 4-10**)

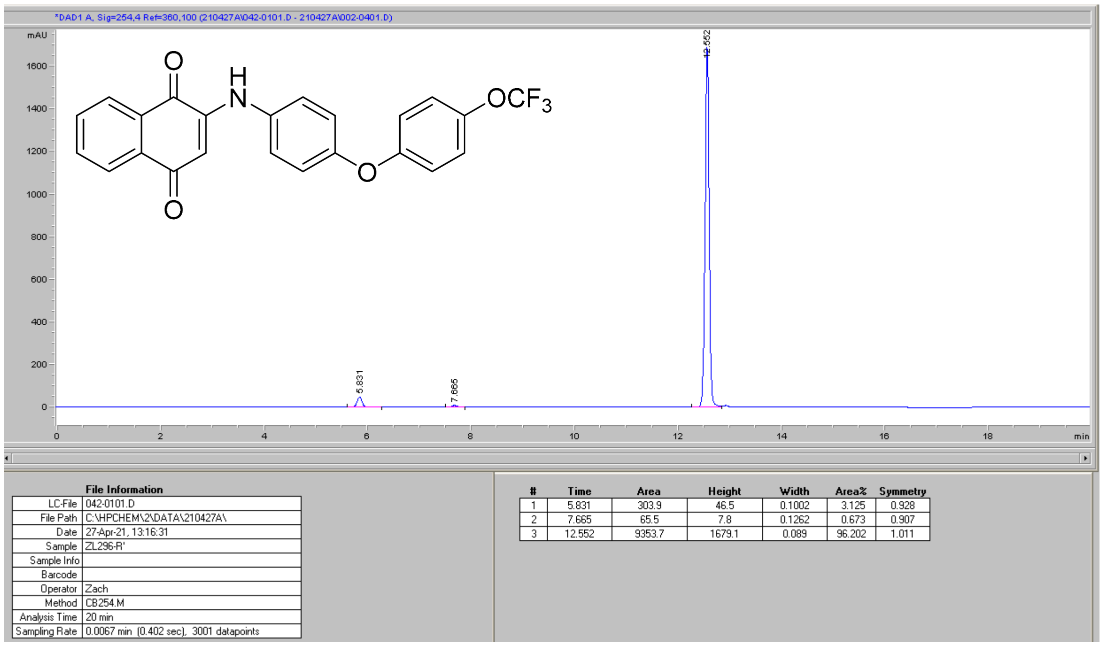

File Information

LC-File	042-0101.D
File Path	C:\HPCHEM\2\DATA\210427A\
Date	27-Apr-21, 13:16:31
Sample	ZL296-R'
Sample Info	
Barcode	
Operator	Zach
Method	CB254.M
Analysis Time	20 min
Sampling Rate	0.0067 min (0.402 sec), 3001 datapoints

#	Time	Area	Height	Width	Area%	Symmetry
1	5.831	303.9	46.5	0.1002	3.125	0.928
2	7.665	65.5	7.8	0.1262	0.673	0.907
3	12.552	9353.7	1679.1	0.089	96.202	1.011

^{1}H NMR of 2-((4-(4-(trifluoromethoxy)phenoxy)phenyl)amino)naphthalene-1,4-dione (**16, 4-10**)

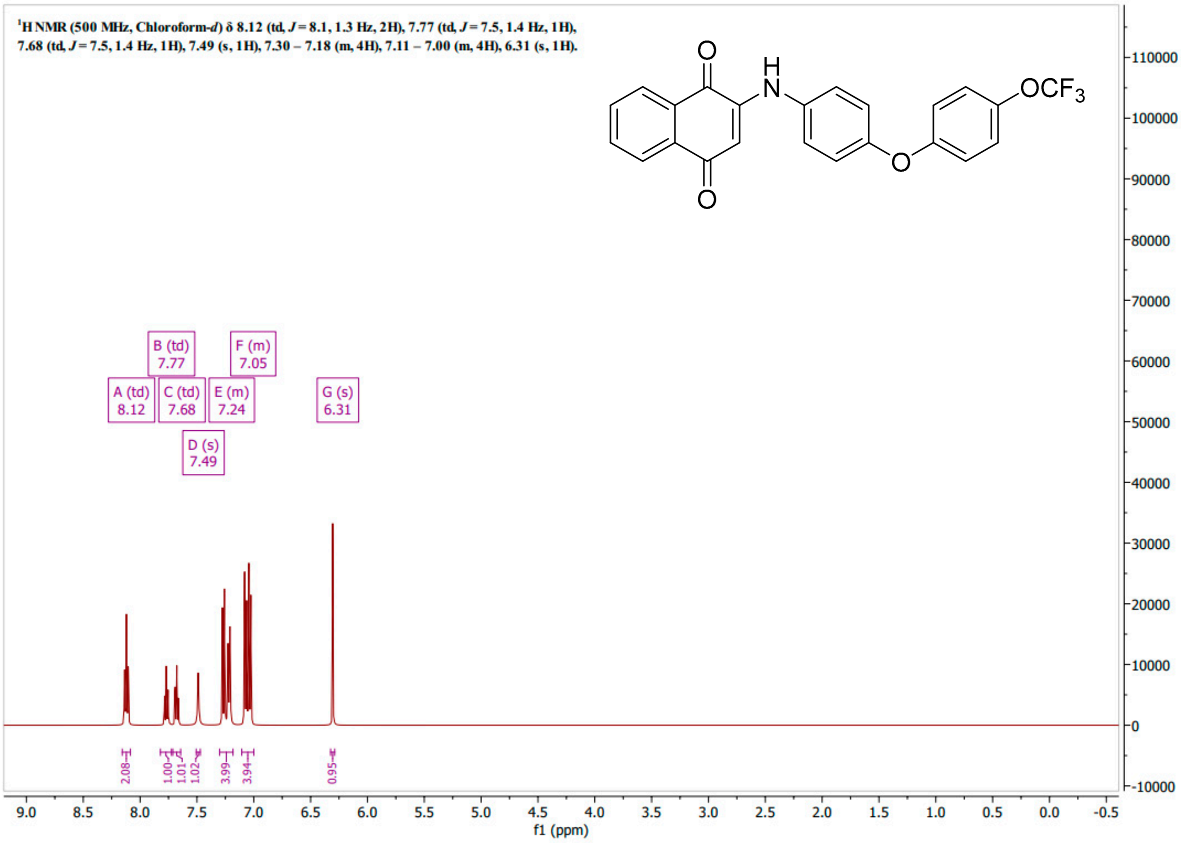

^{13}C NMR of 2-((4-(4-(trifluoromethoxy)phenoxy)phenyl)amino)naphthalene-1,4-dione (**16, 4-10**)

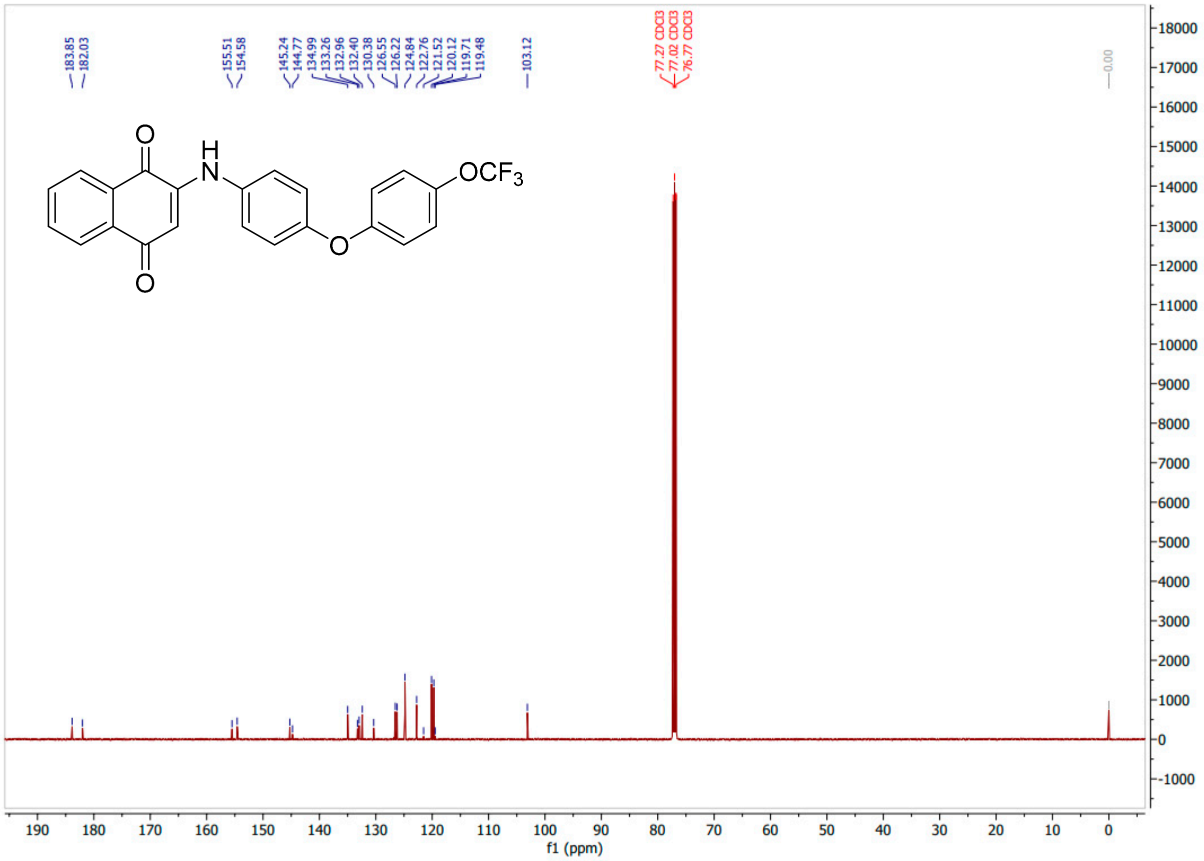

HRMS of 2-((4-(4-(trifluoromethoxy)phenoxy)phenyl)amino)naphthalene-1,4-dione (**16, 4-10**)

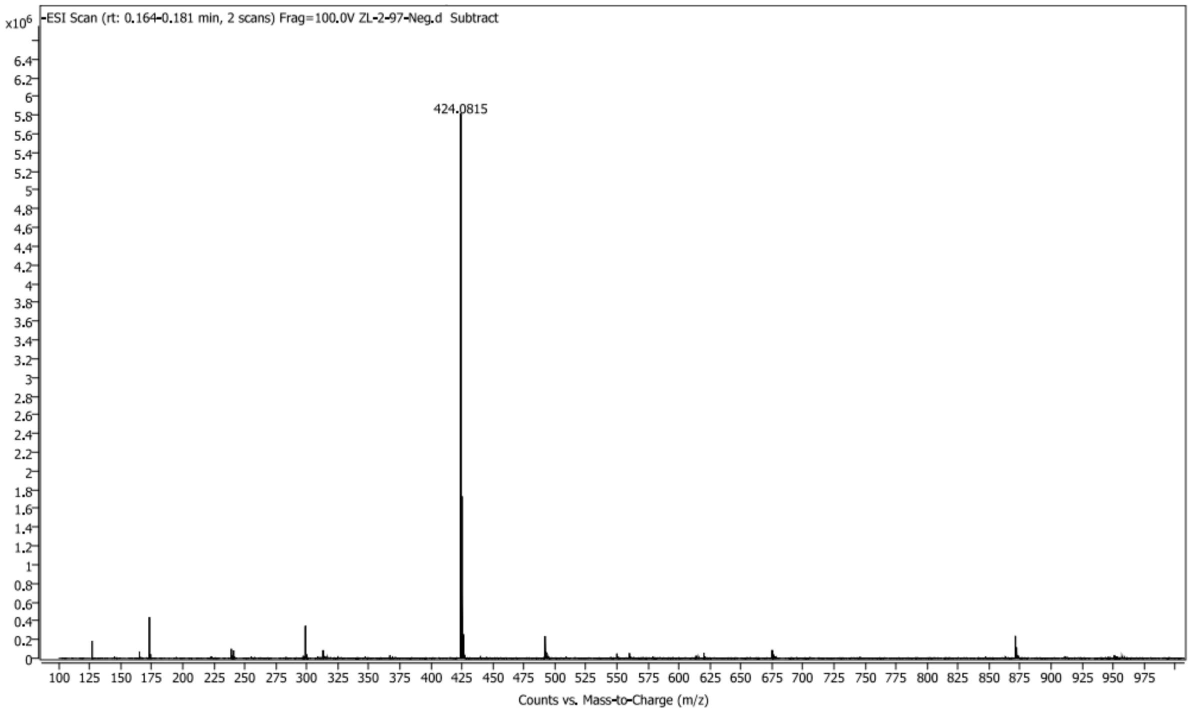

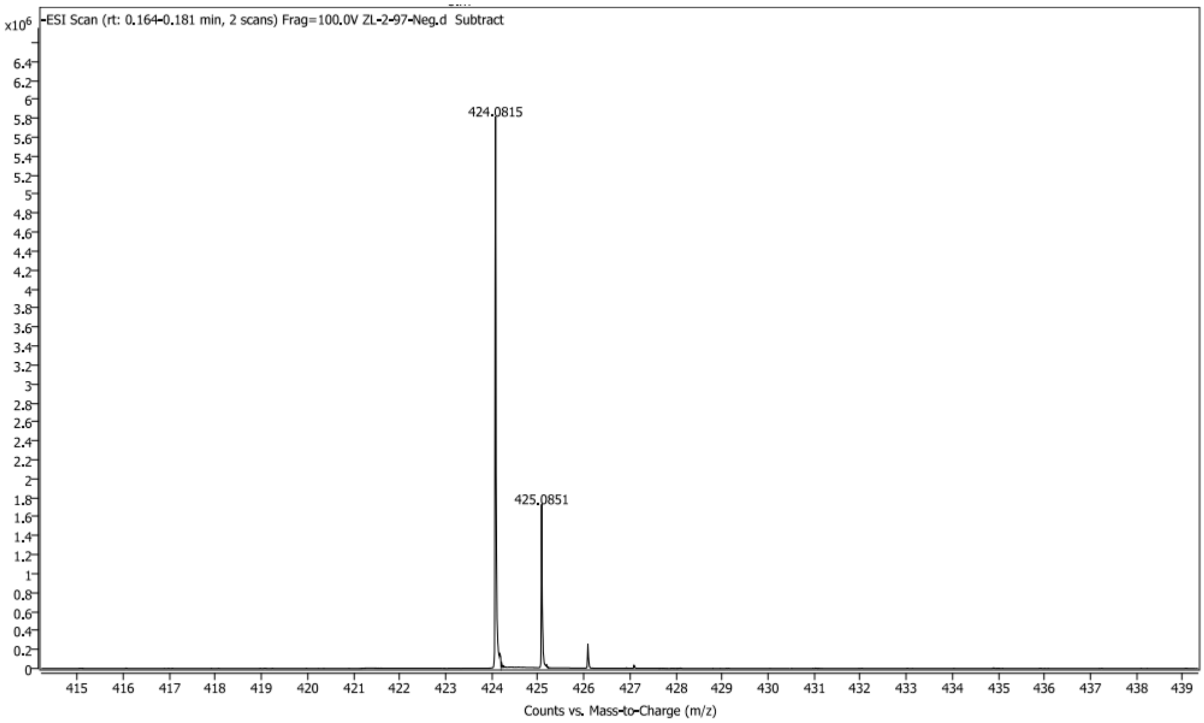

Supplement: Supplementary file 1 [file ijms-27-01198-s001.zip › ijms-4071250-supplementary.pdf]
